# Role of LncSNHG5 in MAFLD: Mechanisms of Arid1a K391 lactylation and lipid accumulation

**DOI:** 10.1002/ctm2.70740

**Published:** 2026-07-24

**Authors:** Xinmiao Li, Feng Jiang, Binbo Fang, Lifan Lin, Jianjian Zheng, Tanzhou Chen

**Affiliations:** ^1^ Department of Clinical Laboratory Key Laboratory of Clinical Laboratory Diagnosis and Translational Research of Zhejiang Province The First Affiliated Hospital of Wenzhou Medical University Wenzhou China; ^2^ Zhejiang Key Laboratory of Intelligent Cancer Biomarker Discovery and Translation The First Affiliated Hospital of Wenzhou Medical University Wenzhou China; ^3^ Department of Clinical Laboratory The Third Affiliated Hospital of Wenzhou Medical University Ruian China; ^4^ Department of Gastroenterology and Hepatology The First Affiliated Hospital of Wenzhou Medical University Wenzhou China

**Keywords:** HBO1, lactylation, LncSNHG5, MAFLD, P300

## Abstract

**Introduction:**

Metabolic dysfunction‐associated fatty liver disease (MAFLD) is a common chronic liver condition marked by abnormal lipid metabolism.

**Objectives:**

Small nucleolar RNA host gene 5 (SNHG5) is involved in the regulation of cell proliferation and apoptosis and was previously identified as a profibrotic factor in liver fibrosis. However, its role in MAFLD remains unclear. This study aimed to elucidate the contribution of SNHG5 to MAFLD progression.

**Methods and results:**

SNHG5 expression was markedly elevated during MAFLD progression, whereas SNHG5 inhibition suppressed lipid accumulation. Transcriptomic sequencing of primary hepatocytes overexpressing SNHG5 demonstrated significant downregulation of monocarboxylate transporter 1 (MCT1), a key lactate transporter. Integrated lactyl‐proteomic and proteomic analyses further revealed that SNHG5 overexpression promoted lactylation of the non‐histone protein AT‐rich interaction domain 1A (Arid1a) at K391, thereby driving excessive lipid accumulation. Acyltransferase assays indicated that both P300 and HBO1 participated in SNHG5‐mediated Arid1a lactylation. In vivo, hepatocyte‐specific SNHG5 knockout markedly attenuated hepatic lipid accumulation and MAFLD progression, whereas simultaneous hepatocyte‐specific deletion of SNHG5 and MCT1 restored lipid accumulation compared with SNHG5‐deficient mice.

**Conclusion:**

SNHG5 promotes lipid accumulation and MAFLD progression through induction of MCT1‐mediated Arid1a K391 lactylation.

## Graphical abstract

1. SNHG5 is significantly upregulated in MAFLD.

2. SNHG5 promotes hepatic lipid accumulation by downregulating MCT1 and regulating intracellular lactate metabolism.

3. SNHG5 drives lactylation of Arid1a at the K391 residue through the P300 and HBO1 acyltransferases.

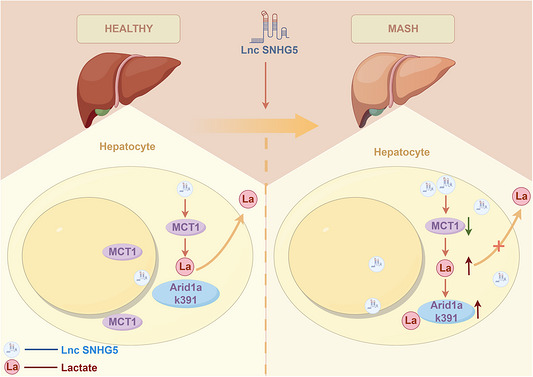

## 1 INTRODUCTION

Metabolic dysfunction‐associated fatty liver disease (MAFLD) is a chronic liver disorder.^1^ Epidemiological studies indicate that at least 20% of patients with MAFLD may progress to metabolic dysfunction‐associated steatohepatitis, which can subsequently develop into cirrhosis and hepatocellular carcinoma (HCC).^2^, ^3^ The primary features of MAFLD include hepatic steatosis, hepatocyte injury, inflammatory cell infiltration and varying degrees of fibrosis.^2^, ^3^, ^4^, ^5^ Prolonged consumption of high‐calorie diets and unhealthy lifestyle behaviours contribute to chronic or excessive fat accumulation, thereby increasing the risk of developing MAFLD.^2^ Analyses covering 2013‒2022 indicate that MAFLD has become a significant contributor to HCC.^6^ Owing to the absence of specific pharmacological therapies, current treatment strategies are largely limited to addressing underlying etiologies and risk factors, with liver transplantation applied in selected cases. These observations highlight the importance of investigating the molecular mechanisms underlying MAFLD to inform potential therapeutic strategies.

Evidence is mounting that long non‐coding RNAs (lncRNAs), which are RNA molecules longer than 200 nucleotides, significantly influence the onset and progression of MAFLD.^7^ lncRNAs modulate chromatin structure and function, as well as transcription of both nearby and distant genes, through interactions with DNA, RNA and proteins, thereby influencing RNA splicing, stability and translation.^7^ Small nucleolar RNA host gene 5 (SNHG5), also known as U50HG, comprises six exons and two small nucleolar RNAs (snoRNAs) (U50 and U50′) encoded within introns 4 and 5, respectively, and is involved in regulating cell proliferation and apoptosis.^8^, ^9^ For example, SNHG5 regulates trophoblast cell proliferation, invasion and migration by modulating the miR‐26a‐5p/N‐cadherin axis.^10^ Our previous research demonstrated high expression of SNHG5 in hepatic stellate cells (HSCs), where it mediates HSC activation through regulation of the NF2 and Hippo pathways.^11^ This regulatory role in HSC activation suggests potential involvement in liver fibrosis, a key pathological stage in MAFLD progression. However, the functional role of SNHG5 in MAFLD remains entirely unknown. Given that MAFLD can progress to liver fibrosis, cirrhosis and HCC, and that SNHG5 is highly expressed and promotes HCC progression,^12^, ^13^ it is plausible that SNHG5 contributes to the pathogenesis and progression of MAFLD, particularly during the transition towards more severe liver disease. Additionally, SNHG5's regulation of inflammation via the NFκB pathway is noteworthy,^13^ as chronic inflammation is a central feature of MAFLD progression towards steatohepatitis. These observations highlight the need for further investigation into the regulatory role of SNHG5 in MAFLD and its downstream mechanisms.

Lactate is a metabolic byproduct of glycolysis and has traditionally been regarded as both an energy source and a metabolic waste product under hypoxic conditions.^14^ Recent studies have reported that dysregulated lactate levels contribute to multiple disease processes, including tumour progression and fibrosis.^15^, ^16^, ^17^ Lactate further influences cellular signalling, gene expression, tumour development and immune metabolism through direct protein interactions and lactylation modifications.^18^, ^19^ Under physiological conditions, monocarboxylate transporter (MCT) subtypes 1–4 serve as the primary lactate transporters,^20^ and previous studies have demonstrated a close association between lactate and MAFLD.^21^ Lactylation is a post‐translational modification (PTM) in which accumulated lactate modifies lysine residues on histones.^14^ Notably, non‐histone proteins can also undergo lactylation.^19^ For example, lactylation of MRE11 at K673 promotes DNA binding and facilitates DNA end resection and homologous recombination.^22^ Lactylation is primarily regulated by lysine acetyltransferases (KATs) and lysine deacetylases (KDACs).^23^, ^24^ KATs include the p300/CBP, GNAT and MYST families; members of the p300/CBP and GNAT families, including GCN5, are established catalysts for multiple acylation types, including lactylation, succinylation and acetylation.^25^ HBO1, a MYST family member, is a multifunctional lysine acetyltransferase capable of catalysing histone lactylation, acetylation, propionylation, butyrylation and crotonylation.^26^ However, the role of HBO1 in regulating non‐histone lactylation remains unclear. Therefore, investigating the regulatory function of HBO1 in non‐histone lactylation may provide new insights into MAFLD pathogenesis and potential therapeutic strategies.

In this study, SNHG5 was found to increase lactate levels by regulating MCT1 and to induce lactylation of AT‐rich interaction domain 1A (Arid1a) at K391, thereby contributing to MAFLD development. Subsequent experiments demonstrated that acetyltransferases P300 and HBO1 mediated SNHG5‐induced lactylation of Arid1a at K391. These findings elucidate a mechanism underlying MAFLD progression and highlight potential therapeutic targets for intervention.

## 2 MATERIALS AND METHODS

### 2.1 Animal

Four‐week‐old mice were fed either a high‐fat diet (HFD) or a high‐fat, high‐cholesterol diet (HFHC) for 16 weeks to establish an MAFLD mouse model, while a control group received a normal diet (ND). At the end of the feeding period, blood, liver tissue and primary hepatocytes were collected for subsequent analyses. The HFD provided 60% kcal from fat, 20% kcal from carbohydrates and 20% kcal from protein. The HFHC diet consisted of 40% fat, 20% fructose, 18% protein, 2% cholesterol and 20% other carbohydrates by weight.

Hepatocyte‐specific SNHG5‐knockout mice (*SNHG5^–/–^*) were generated by crossing SNHG5^flox/flox^ mice with Alb‐Cre^+^ mice. To generate hepatocyte‐specific SNHG5 and MCT1 double‐knockout (DKO) mice, we first crossed SNHG5^flox/flox^ mice with MCT1^flox/flox^ mice to produce double heterozygous offspring (SNHG5^flox/+^, MCT1^flox/+^). These double heterozygotes were intercrossed to obtain SNHG5^flox/flox^ and MCT1^flox/flox^ double‐floxed mice. Subsequently, double‐floxed mice were crossed with Alb‐Cre transgenic mice. Offspring genotyped as SNHG5^flox/flox^, MCT1^flox/flox^ and Alb‐Cre^+^ were designated as DKO mice, while littermates lacking the Alb‐Cre transgene served as controls (Cyagen).

All animal experiments were performed in accordance with the ARRIVE Guidelines for Reporting In Vivo Experiments and the Guidelines for the Management and Use of Laboratory Animals of Wenzhou Medical University. The study protocol was approved by the Laboratory Animal Ethics Committee of Wenzhou Medical University (approval number: wydw2024‐0443).

### 2.2 AAV8‐mediated hepatocyte‐specific overexpression of SNHG5 in mice

Recombinant AAV8 vectors carrying mouse SNHG5 under the hepatocyte‐specific albumin promoter and empty AAV8 vectors (control) were purchased from Cyagen (Suzhou).

### 2.3 Human specimens

Serum samples from human subjects were collected from patients receiving treatment at the First Affiliated Hospital of Wenzhou Medical University. All participants gave written informed consent, and the study complied with the Declaration of Helsinki ethical guidelines. The study protocol was approved by the Ethics Committee of the First Affiliated Hospital of Wenzhou Medical University on 18 November 2021 (approval number: KY2022‐139).

### 2.4 Serum alanine aminotransferase and aspartate aminotransferase assay

Serum levels of alanine aminotransferase (ALT) (Cat. No. C009‐2‐1) and aspartate aminotransferase (AST) (Cat. No. C010‐2‐1) in mice were measured using assay kits (Nanjing Jiancheng Bioengineering Institute). Based on enzymatic colorimetry, enzyme activity was calculated by comparing the absorbance of samples with standard calibration solutions. Serum was isolated by centrifugation, and all operations were conducted strictly following the kit instructions.

### 2.5 Staining of liver tissues

Liver sections were fixed routinely, then stained using Oil Red O (Servicebio, Cat. No. 2505E032), Nile Red (Servicebio, Cat. No. GDP1036), haematoxylin‒eosin (HE) (Servicebio, Cat. No. G1005), Masson (Servicebio, Cat. No. GP1032) and Sirius Red (Servicebio, Cat. No. G1078) stain kits, respectively. After color differentiation and rinsing, all sections were dehydrated, cleared and mounted for microscopic observation.

### 2.6 Primary hepatocytes isolation

Hepatocytes were isolated from mice across different experimental groups using a two‐step collagenase perfusion technique. Mice were anaesthetised, and livers were perfused via the portal vein with perfusion solution to remove blood, followed by infusion of a collagenase mixture (GlpBio, Cat. No. GC19591). The resulting cell suspension was filtered through a 70‐µm strainer and centrifuged to isolate hepatocytes, which were subsequently cultured on collagen‐coated plates.

### 2.7 Cell culture

Primary hepatocytes from C57BL/6 mice were cultured in specialised complete medium (Shenzhen LV Bio Tech, Cat. No. WEM006). The AML12 cell line was obtained from Newgainbio (Wuxi) and maintained in complete medium (Shanghai iCell Bioscience Inc., Cat. No. M003‐001b) at 37°C with 5% CO_2_. For lipid accumulation induction, hepatocytes were treated with a mixture of palmitic acid (.25 mmol/L) and oleic acid (.5 mmol/L) (PO mixture) (Kunchuang Technology, Cat. No. KC006).

### 2.8 Construction of cell lines with overexpression

SNHG5 and MCT1 overexpression plasmids were constructed in the pcDNA3.1 backbone, with empty pcDNA3.1 vectors used as controls. Transfection was performed using Lipofectamine 3000 reagent (Invitrogen, Cat. No. L3000015) according to the manufacturer's protocol. Briefly, primary hepatocytes and AML12 cells were seeded, and complexes of plasmid DNA and Lipofectamine 3000 were added to the culture medium. Cells were incubated for 48 h before being harvested for downstream analyses.

### 2.9 Quantitative real‐time PCR

Total RNA was isolated with the RNA Extraction Kit (TIANGEN, Cat. No. DP419) following the manufacturer's protocols. RNA purity and concentration were assessed with the Thermo Fisher NanoDrop 2000. Total RNA was reverse‐transcribed into cDNA using the SuperScript IV Reverse Transcription Kit (Thermo Fisher, Cat. No. 18091050) according to the manufacturer's instructions. Quantitative PCR was executed on a real‐time PCR system utilising SYBR Green qPCR Master Mix (Takara, Cat. No. RR820A). β‐Actin was served as the internal reference gene. Relative mRNA expression was calculated using the 2^−ΔΔCt^ method. Primer sequences are provided in Table S1.

### 2.10 RNA sequencing profiling

Total RNA from primary hepatocytes of Vector and SNHG5‐overexpressing groups was isolated and quality‐checked. RNA sequencing libraries were constructed, and sequencing was performed on the Illumina NovaSeq 6000 platform. Differential expression analysis was conducted using a threshold of |log_2_ fold change| > .585 and adjusted *p* < .05.

Subsequent Kyoto Encyclopedia of Genes and Genomes (KEGG) pathway enrichment analysis identified glycolysis/gluconeogenesis as the most significantly enriched pathway in SNHG5‐overexpressing cells. Genes related to glucose metabolic homeostasis were next selected for heatmap visualisation and further study.

### 2.11 Proteome and lactylome analysis

Proteome and lactylome profiling, as well as integrated analyses, were conducted by Shanghai Zhongke New Life Biotechnology Co., Ltd. Proteins or modifications with |log_2_ fold change| > 1.5 and *p* < .05 were considered differentially expressed.

Data‐independent acquisition (DIA) datasets were processed using Spectronaut software, with trypsin as the enzyme (maximum of two missed cleavages allowed). Fixed modifications included carbamidomethylation on cysteine (C), while dynamic modifications included methionine oxidation (M), N‐terminal acetylation and lysine lactylation (K). Protein and peptide identifications were validated with a false discovery rate ≤ 1%.

### 2.12 RNA pull‐down and liquid chromatography‒tandem mass spectrometry analysis

Using the Transcript Aid T7 High Yield Transcription Kit (Thermo Fisher Scientific, Cat. No. K0441), biotinylated SNHG5 was synthesised. Using the Pierce Magnetic RNA‐Protein Pull‐Down Kit (Thermo Fisher Scientific, Cat. No. 20164), SNHG5 binding proteins were captured. Eluted proteins were digested with trypsin and analysed by liquid chromatography‒tandem mass spectrometry (LC‒MS/MS) at GuangK Ande Biotechnology Co., Ltd.

### 2.13 Western blot

Electrophoresis on 10% sodium dodecyl sulfate (SDS)‒polyacrylamide gels was used to separate protein extracts, which were then transferred to polyvinylidene difluoride (PVDF) membranes. Specific primary antibodies were used to incubate the membranes, and enhanced chemiluminescence was employed to detect protein bands. Normalisation was achieved using β‐actin as the internal control, and ImageJ software was employed for protein expression quantification. Refer to Table S2 for antibody sources.

### 2.14 Statistical analysis

All statistical calculations were conducted via GraphPad Prism 10. Two‐group comparisons were analysed by Student's *t*‐test, whereas one‐way ANOVA was adopted for comparisons involving three or more cohorts. Statistical significance was defined as *p* < .05.

## 3 RESULTS

### 3.1 SNHG5 expression is increased in MAFLD both in vivo and in vitro

**FIGURE 1:**
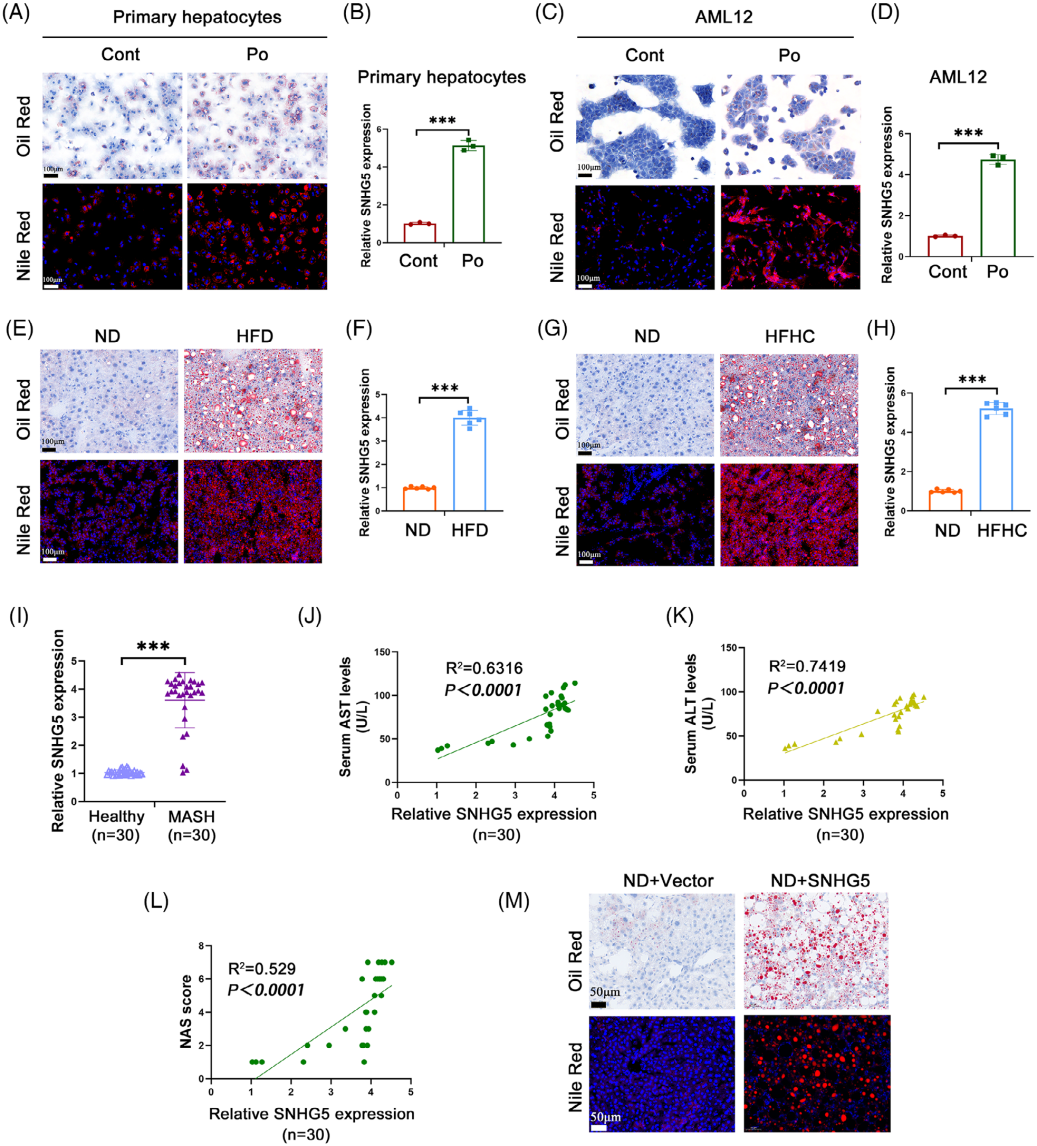
Small nucleolar RNA host gene 5 (SNHG5) expression is elevated in metabolic dysfunction‐associated fatty liver disease (MAFLD) both in vivo and in vitro PO mixture: palmitic acid ( .25 mmol/L) and oleic acid (.5 mmol/L). (A and C) Oil Red O and Nile Red staining in primary hepatocytes and AML12 cells (*n* = 3). (B and D) Quantitative real‐time PCR (qRT‐PCR) analysis of SNHG5 expression in primary hepatocytes and AML12 cells (*n* = 3). (E and G) Oil Red O and Nile Red staining in liver tissues (*n* = 6). (F and H) qRT‐PCR analysis of SNHG5 expression in liver tissues (*n* = 6). (I) qRT‐PCR analysis of SNHG5 expression in serum samples (*n* = 30). (J and K) Linear regression analysis of the correlation between SNHG5 expression and serum aspartate aminotransferase (AST) or alanine aminotransferase (ALT) levels in patients with metabolic dysfunction‐associated steatohepatitis (MASH) (*n* = 30). (L) Linear regression analysis of the correlation between SNHG5 expression and NAS score. (M) Representative images of Oil Red O and Nile Red staining in liver tissues transfected with empty vector (ND + Vector) or SNHG5 overexpression vector (ND + SNHG5). ^***^ *p* < .001.

To investigate the role of SNHG5 in MAFLD, we first validated the successful establishment of MAFLD mouse models. In HFD‐ and HFHC‐fed mice, macroscopic liver morphology (Figure S1A), Oil Red O staining (Figure 1E,G) and HE staining (Figure S1D) revealed marked hepatic steatosis compared with ND controls. Correspondingly, liver weight (Figure S1B), hepatic triglyceride (TG) content (Figure S1C), and serum levels of ALT and AST (Figure S1E) were significantly elevated, confirming the successful modelling of MAFLD in vivo. Oil Red O and Nile Red staining further demonstrated effective lipid droplet accumulation in primary hepatocytes and AML12 cells, indicating successful in vitro MAFLD modelling (Figure 1A,C). Quantitative real‐time PCR (qRT‐PCR) analysis showed that SNHG5 expression was upregulated in cultured primary hepatocytes and AML12 cells, as well as in liver tissues from HFD‐ and HFHC‐fed mice (Figure 1B,D,F,H). Moreover, SNHG5 expression was elevated in serum from patients with MAFLD compared with healthy controls, and positively correlated with liver injury markers (ALT and AST) and NAS score (Figure 1I–L). Hepatocyte‐specific SNHG5 overexpression in mice significantly increased hepatic lipid accumulation, as indicated by Oil Red O and Nile Red staining (Figure 1M). These results suggest that elevated SNHG5 expression is associated with MAFLD occurrence and progression.

### 3.2 SNHG5 deficiency inhibits lipid deposition, inflammation and fibrosis in vitro and in vivo

**FIGURE 2:**
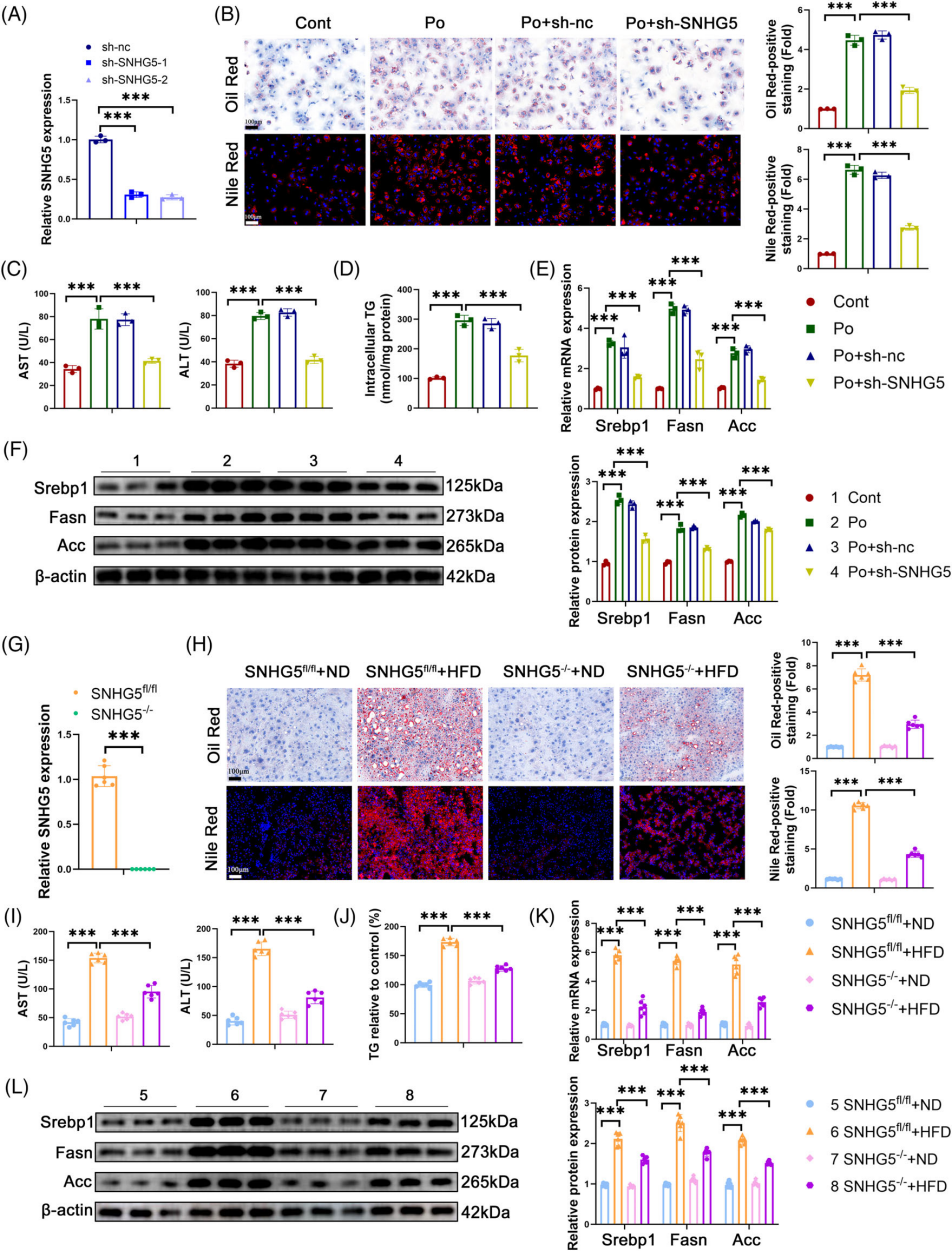
Small nucleolar RNA host gene 5 (SNHG5) deficiency suppresses lipid deposition in vitro and in vivo. (A) Quantitative real‐time PCR (qRT‐PCR) analysis of SNHG5 expression in primary hepatocytes (*n* = 3). (B) Oil Red O and Nile Red staining in primary hepatocytes. (C) Aspartate aminotransferase (AST) and alanine aminotransferase (ALT) levels in the culture supernatant of primary hepatocytes (*n* = 3). (D) Intracellular triglyceride (TG) levels in primary hepatocytes (*n* = 3). (E and F) qRT‐PCR and Western blot analysis of Srebp1, Fasn and Acc mRNA and protein expression in primary hepatocytes (*n* = 3). (G) qRT‐PCR analysis of SNHG5 expression in liver tissues (*n* = 6). (H) Oil Red O and Nile Red staining in liver tissues (*n* = 6). (I) Serum AST and ALT levels (*n* = 6). (J) Liver TG content relative to control (*n* = 6). (K and L) qRT‐PCR and Western blot analysis of Srebp1, Fasn and Acc mRNA and protein expression in liver tissues (*n* = 6). ^***^ *p* < .001.

To assess the functional role of SNHG5 in MAFLD progression, short hairpin RNA was used to generate SNHG5‐knockdown models in primary hepatocytes and AML12 cells (Figures 2A and S2A). Oil Red O and Nile Red staining demonstrated that sh‐SNHG5 significantly inhibited PO‐induced lipid droplet accumulation in primary hepatocytes (Figure 2B) and AML12 cells (Figure S2B). SNHG5 knockdown also attenuated PO‐induced liver injury, as reflected by reduced AST and ALT levels in the culture supernatant of primary hepatocytes (Figure 2C) and AML12 cells (Figure S2C). MAFLD progression involves both exogenous fatty acid uptake and hepatic de novo lipogenesis. Here, we used a PO‐induced fatty acid overload model to test whether SNHG5 regulates lipid accumulation, we examined whether SNHG5 modulates this process. sh‐SNHG5 reduced intracellular TG content in primary hepatocytes (Figure 2D) and AML12 cells (Figure S2D). At the molecular level, SNHG5 knockdown downregulated the mRNA and protein expression of fatty acid synthesis‐related genes, including Srebp1, Fasn and Acc, in both primary hepatocytes (Figure 2E,F) and AML12 cells (Figure S2E,F). To confirm the in vivo function of SNHG5, SNHG5^–/–^ mice were generated, and both HFD‐ and HFHC‐induced MAFLD models were established (Figure 2G). Oil Red O and Nile Red staining showed that SNHG5 knockout markedly reduced lipid droplet accumulation in the livers of HFD‐fed mice (Figure 2H) and HFHC‐fed mice (Figure S2G). Consistently, SNHG5 deficiency significantly decreased serum ALT and AST levels (Figures 2I and S2H) and hepatic TG content (Figures 2J and S2I). Additionally, mRNA and protein expression of Srebp1, Fasn and Acc were downregulated in livers of SNHG5^–/–^ mice in both diet models (Figures 2K,L and S2J,K). Given the close association of MAFLD progression with inflammation and fibrosis, we evaluated the effects of SNHG5 knockout on these pathological features in HFD‐fed mice. qRT‐PCR analysis revealed that SNHG5 deficiency significantly reduced hepatic mRNA levels of pro‐inflammatory cytokines tumour necrosis factor‐α and interleukin‐6 (Figure S3A). Furthermore, expression of fibrosis markers α‐SMA and Col1a1 was markedly decreased in SNHG5^–/–^ mice compared with SNHG5*fl/fl* littermates (Figure S3B). In summary, these results demonstrate that SNHG5 deficiency inhibits lipid accumulation and fatty acid synthesis in vitro and ameliorates hepatic steatosis, inflammation, and fibrosis in vivo.

### 3.3 SNHG5 promotes lipid accumulation by regulating lactate via MCT1

**FIGURE 3:**
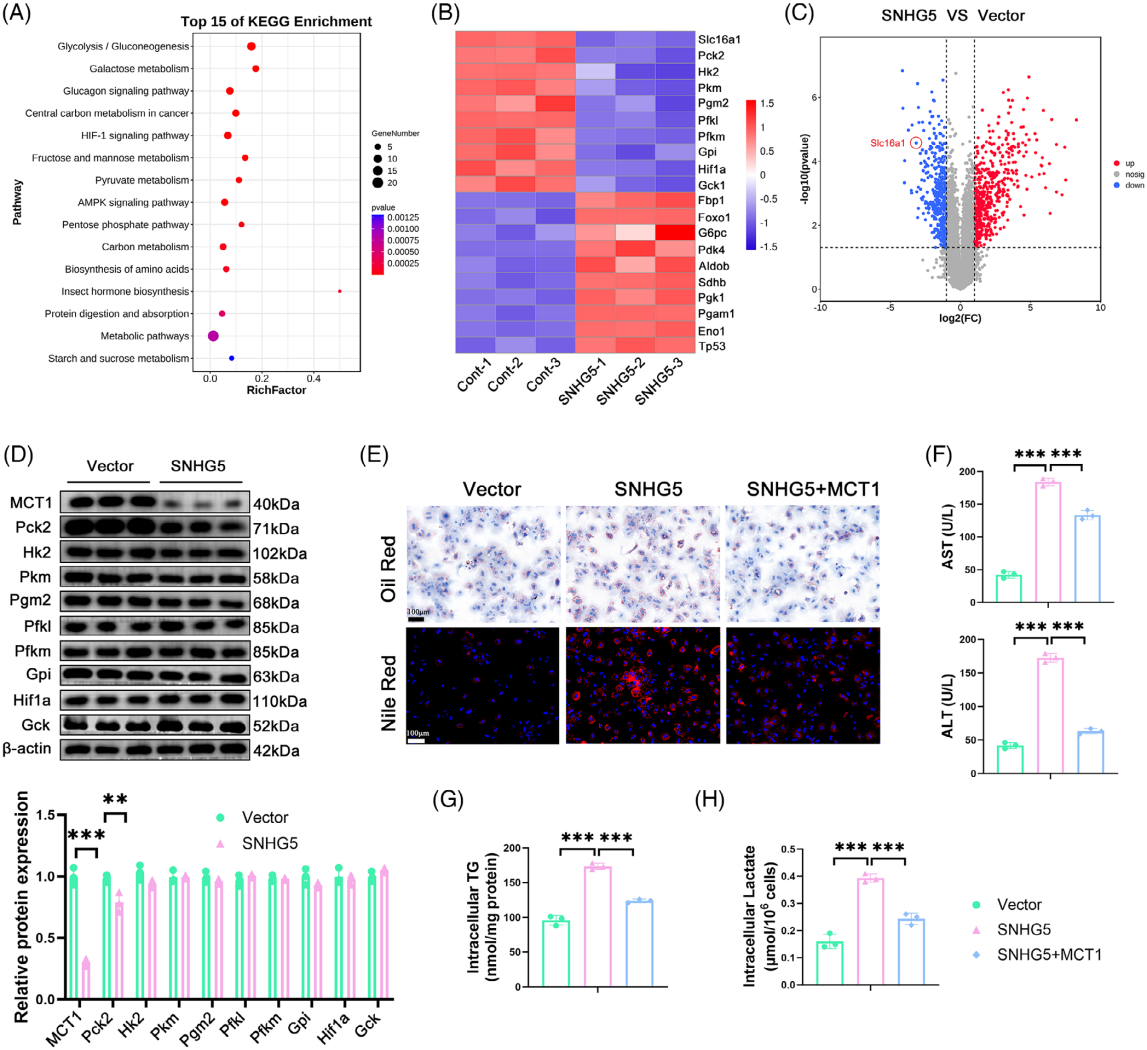
Small nucleolar RNA host gene 5 (SNHG5) promotes lipid accumulation through regulation of lactate via MCT1. (A) Kyoto Encyclopedia of Genes and Genomes (KEGG) enrichment analysis of differentially expressed genes (DEGs) from RNA sequencing of primary hepatocytes. (B) Heatmap showing expression profiles of DEGs involved in glucose metabolic homeostasis in primary hepatocytes. (C) Volcano plot of DEGs in primary hepatocytes. (D) Western blot analysis of MCT1 and key glycolysis‐related proteins, including Pck2, Hk2, Pkm, Pgm2, Pfkl, Pfkm, Gpi, Hif1a and Gck, in primary hepatocytes. (E) Oil Red O and Nile Red staining in primary hepatocytes. (F) Aspartate aminotransferase (AST) and alanine aminotransferase (ALT) levels in the culture supernatant of primary hepatocytes. (G) Intracellular triglyceride (TG) content in primary hepatocytes. (H) Intracellular lactate levels in primary hepatocytes. *n* = 3, ^***^ *p* < .001.

To further investigate the mechanisms by which SNHG5 mediates MAFLD progression, RNA sequencing was performed on primary hepatocytes overexpressing SNHG5. KEGG pathway enrichment and heatmap analyses revealed that differentially expressed genes were associated with pathways including glycolysis/gluconeogenesis, HIF‐1 signalling and broader metabolic pathways, with glycolysis being the most significantly affected (Figure 3A,B). Subsequent volcano plot analysis of glycolysis‐related genes highlighted Slc16a1, which encodes MCT1, as significantly downregulated (Figure 3C). Western blot analysis confirmed that SNHG5 overexpression reduced protein levels of key glycolysis‐related molecules (these molecules correspond to the genes significantly downregulated in the heatmap), with MCT1 showing the most pronounced decrease (Figure 3D). Accordingly, MCT1 was selected for further functional studies. Next, the role of MCT1 in SNHG5‐mediated MAFLD progression was explored. Functional assays demonstrated that MCT1 overexpression significantly reversed SNHG5‐induced lipid droplet accumulation in primary hepatocytes (Figure 3E) and AML12 cells (Figure S4A), as assessed by Oil Red O and Nile Red staining. MCT1 overexpression also mitigated SNHG5‐mediated increases in liver injury markers (ALT and AST; Figures 3F and S4B), intracellular TG content (Figures 3G and S4C) and lactate levels (Figures 3H and S4D). At the molecular level, MCT1 overexpression downregulated mRNA (Figure S4E) and protein (Figure S4F) levels of fatty acid synthesis‐related genes. Consistently, in vivo overexpression of MCT1 suppressed hepatic lipid deposition (Figure S4G) and reduced Srebp1, Fasn and Acc expression (Figure S4H,K), serum ALT and AST (Figure S4I) and liver TG content (Figure S4J). Furthermore, SNHG5 overexpression did not significantly alter protein levels of other lactate transporters, including MCT2, MCT3 and MCT4 (Figure S4L), indicating that SNHG5 specifically regulates lactate metabolism via MCT1. These results suggest that SNHG5 promotes lipid accumulation by modulating lactate transport through MCT1.

### 3.4 SNHG5 promotes lipid droplet formation by interacting with MCT1 in vitro

**FIGURE 4:**
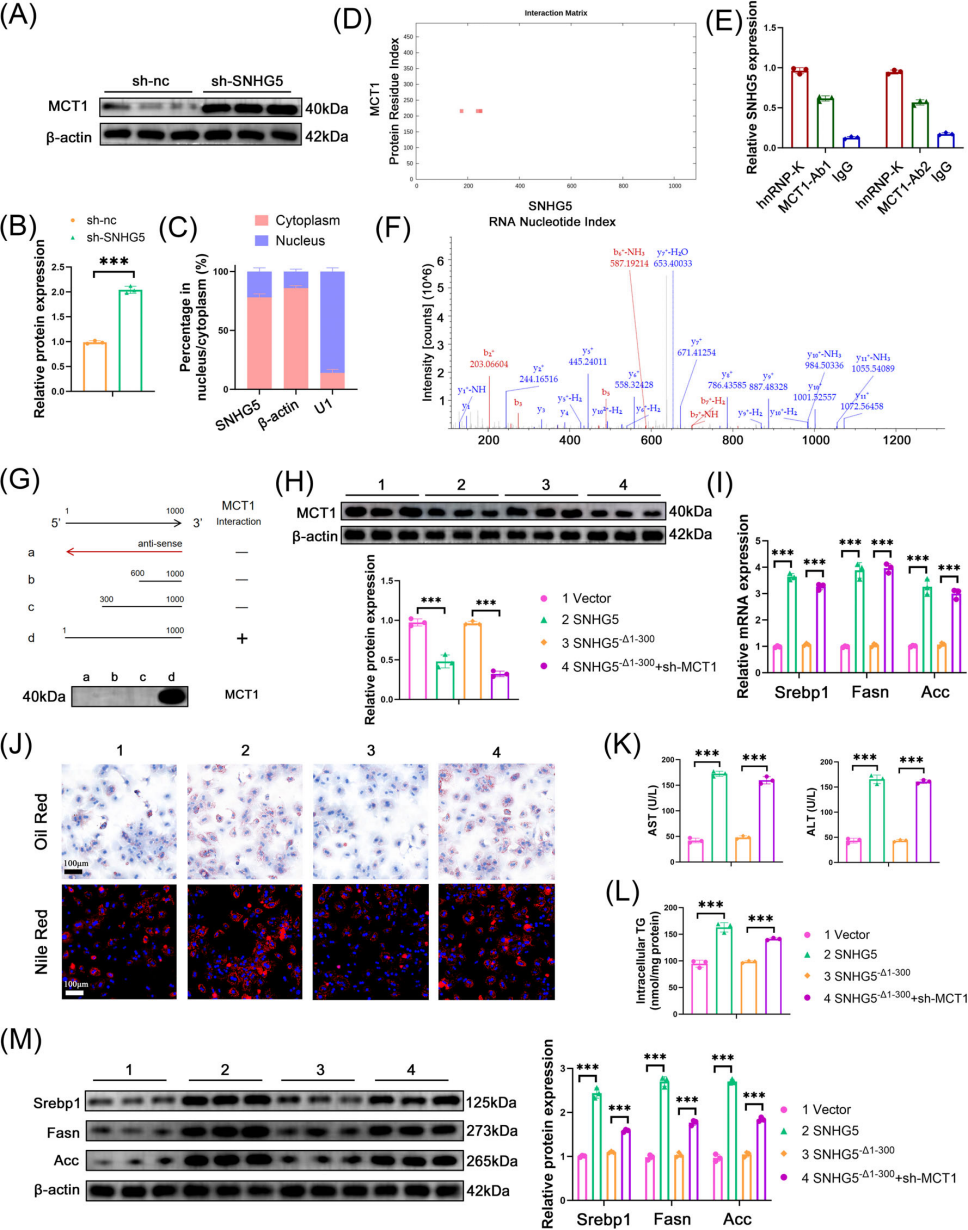
Small nucleolar RNA host gene 5 (SNHG5) promotes lipid droplet formation by interacting with MCT1 in vitro. (A and B) MCT1 protein expression in primary hepatocytes. (C) Nuclear‐cytoplasmic distribution of SNHG5 in primary hepatocytes. (D) catRAPID prediction of the overall interaction propensity between SNHG5 and MCT1 protein. (E) RNA immunoprecipitation (RIP) experiments in primary hepatocytes using MCT1 antibody, with hnRNP‐K antibody and IgG serving as positive and negative controls, respectively. (F) Liquid chromatography‒tandem mass spectrometry (LC‒MS/MS) analysis was performed on RNA pull‐down samples from primary hepatocytes overexpressing SNHG5. (G) Mapping of the interactive region between SNHG5 and MCT1. Biotinylated RNAs corresponding to distinct SNHG5 fragments or their antisense sequences (negative controls) were incubated with cell lysates, and the associated MCT1 protein was subsequently detected. (H) MCT1 protein expression in primary hepatocytes. (I and M) mRNA and protein expression levels of Srebp1, Fasn and Acc in primary hepatocytes. (J) Oil Red O and Nile Red staining in primary hepatocytes. (K) Aspartate aminotransferase (AST) and alanine aminotransferase (ALT) levels in the culture supernatant of primary hepatocytes. (L) Intracellular triglyceride (TG) levels in primary hepatocytes. *n* = 3, ^***^ *p* < .001.

Given that lncRNAs often exert their functions via interactions with RNA‐binding proteins, we next investigated whether SNHG5 regulates MAFLD progression by directly interacting with MCT1. SNHG5 knockdown increased MCT1 protein expression in primary hepatocytes (Figure 4A,B). Subcellular fractionation PCR analysis revealed that SNHG5 is predominantly localised in the cytoplasm, with U1 snRNA and β‐actin serving as nuclear and cytoplasmic markers, respectively (Figure 4C). Bioinformatic prediction using catRAPID indicated a high probability of interaction between the 0 and 300 nt region of SNHG5 and MCT1 protein (Figure 4D), which was subsequently validated by RNA immunoprecipitation (RIP) assays using two independent anti‐MCT1 antibodies (Figure 4E). To validate the interaction between SNHG5 and MCT1, we performed RNA pull‐down coupled with LC‒MS/MS in primary hepatocytes overexpressing SNHG5. Mass spectrometric analysis identified multiple high‐confidence peptide fragments of MCT1 (SLC16A1) in the SNHG5 pull‐down complex (Figure 4F). Deletion mapping confirmed that the 0–300 nt region of SNHG5 is required for binding MCT1 (Figure 4G). To functionally validate this interaction, a deletion mutant SNHG5 (SNHG5^−Δ1‐300^), lacking the 0–300 nt MCT1‐binding region, was constructed. Unlike wild‐type (WT) SNHG5, SNHG5^−Δ1‐300^ failed to downregulate MCT1 protein expression (Figure 4H) and did not enhance lipid droplet accumulation in primary hepatocytes, as shown by Oil Red O and Nile Red staining. Importantly, knockdown of MCT1 restored lipid droplet accumulation (Figure 4J). Consistently, SNHG5^−Δ1‐300^ neither upregulate mRNA or protein levels of fatty acid synthesis‐related genes (Figure 4I,M) nor did it increase liver injury markers (AST and ALT; Figure 4K) or intracellular TG content (Figure 4L). These results demonstrate that the direct interaction between SNHG5 (0–300 nt region) and MCT1 is indispensable for SNHG5‐mediated lipid droplet formation and MAFLD progression in vitro.

### 3.5 SNHG5 promotes Arid1a K391 lactylation

**FIGURE 5:**
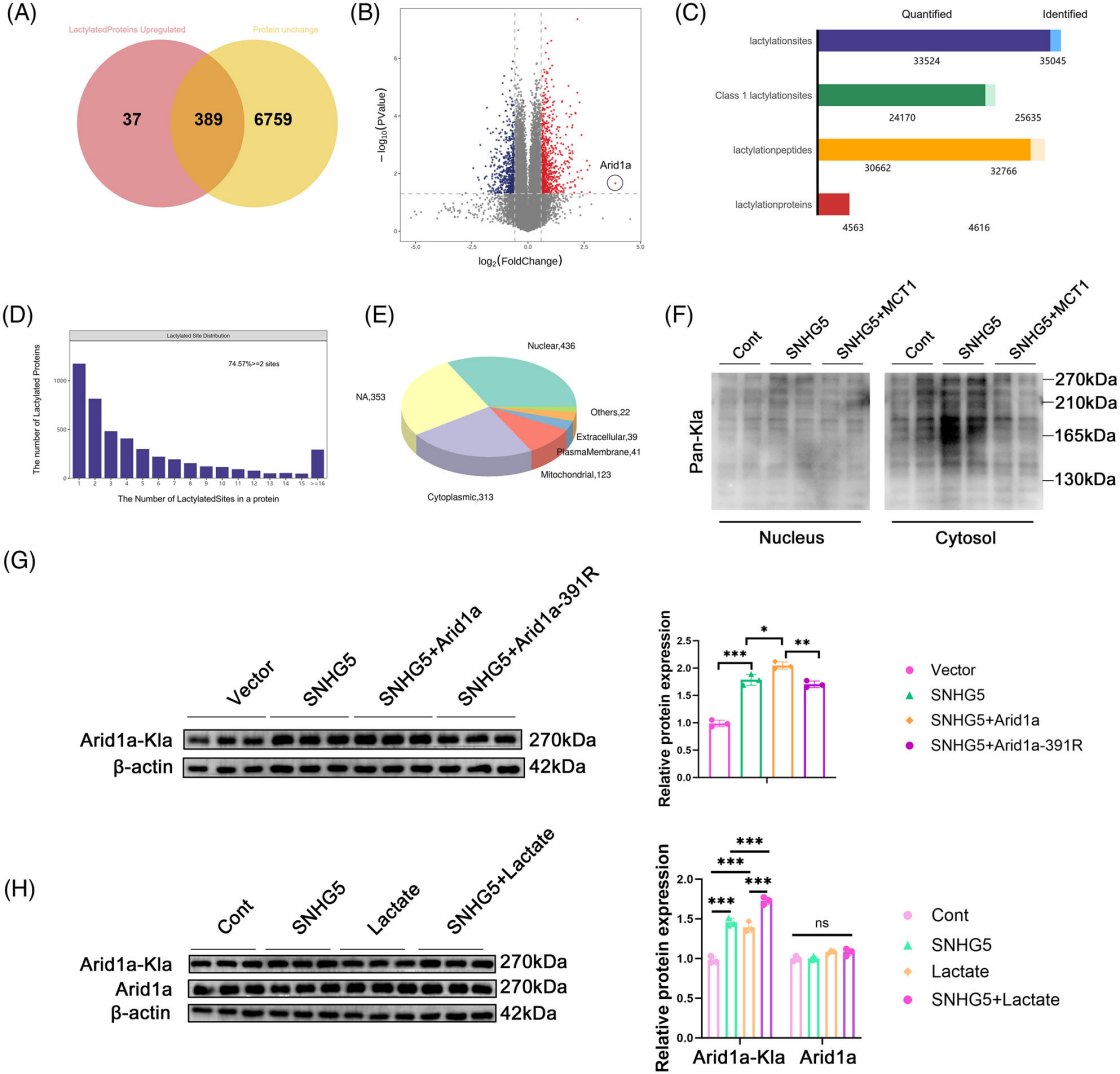
Small nucleolar RNA host gene 5 (SNHG5) promotes Arid1a K391 lactylation. (A) Venn diagram of proteins exhibiting upregulated lactylation with unchanged total protein expression in primary hepatocytes. (B) Volcano plot showing lactylated proteins in primary hepatocytes. (C) Quantification of lactylated peptides, total lactylation sites and high‐confidence Class 1 lactylation sites in primary hepatocytes. (D) Distribution of the number of lactylated sites per protein in primary hepatocytes. (E) Subcellular localisation of proteins with differentially expressed lactylated peptides in primary hepatocytes. (F) Pan‐Kla levels in nuclear and cytosolic fractions of primary hepatocytes. (G) Arid1a‐Kla protein expression levels in primary hepatocytes. (H) Arid1a‐Kla and total Arid1a protein levels in primary hepatocytes. *n* = 3, ^*^ *p* < .05, ^**^ *p* < .01, ^***^ *p* < .001, ns = not significant.

Lactate accumulation promotes protein lactylation, in which lactate covalently binds to lysine residues on target proteins, thereby affecting their structure and function. We observed that SNHG5 overexpression significantly reduced MCT1 expression, coinciding with a substantial increase in intracellular lactate levels.^27^ Given that lactate serves as a substrate for protein lactylation, we hypothesised that SNHG5 downstream mechanisms may involve lactylation. To test this, integrated proteome and lactylome analyses were performed in control and SNHG5‐overexpressing primary hepatocytes. A Venn diagram intersecting proteins with upregulated lactylation modifications and proteins with unchanged total proteome expression identified 389 candidate genes (Figure 5A). Among these, Arid1a exhibited the most significant increase in lactylation (Figure 5B) and was selected for further investigation. Arid1a, a component of the chromatin remodelling complex, has been reported to promote MAFLD development through epigenetic regulation.^28^ To characterise the lactylation landscape, lactylation sites across all identified proteins were quantified. A total of 33 524 quantifiable lactylation sites were detected, including 24 170 high‐confidence Class 1 sites (Figure 5C). Further analysis revealed that 74.57% of lactylated proteins harboured two or more Class 1 lactylation sites (Figure 5D). Subcellular localisation analysis of proteins with differentially modified peptides indicated predominant localisation in the nucleus (436 proteins) and cytoplasm (313 proteins) (Figure 5E), suggesting that SNHG5 may regulate lactylation in both compartments. To identify specific lysine residues on Arid1a undergoing lactylation, lactylome data were analysed. K391 and K985 exhibited the most significant upregulation in the SNHG5‐overexpressing group (Table S3). Both residues were mutated to arginine (R) and introduced into cells via lentiviral transduction. Overexpression of WT Arid1a significantly enhanced Arid1a lactylation (Figure 5G). In contrast, mutation of K985 (Arid1a‐K985R) did not significantly affect Arid1a lactylation levels (Figure S5A,B), indicating that K391 is the primary lactylation site regulated by SNHG5. Subcellular fractionation followed by pan‐lactylation (Pan‐Kla) Western blotting showed that SNHG5 overexpression increased lactylation predominantly in the cytoplasm, and this effect was reversed by MCT1 overexpression (Figure 5F). Consistent with the omics data, Western blot analysis confirmed that SNHG5 overexpression significantly increased Arid1a lactylation (Arid1a‐Kla), while total Arid1a protein levels remained unchanged. Supplementation with exogenous lactate further enhanced Arid1a‐Kla, and this effect was additive with SNHG5 overexpression (Figure 5H). These results demonstrate that SNHG5 promotes Arid1a lactylation at the K391 residue, likely via intracellular lactate accumulation mediated by MCT1 downregulation.

### 3.6 SNHG5 promotes lipid synthesis by promoting Arid1a lactylation at K391

**FIGURE 6:**
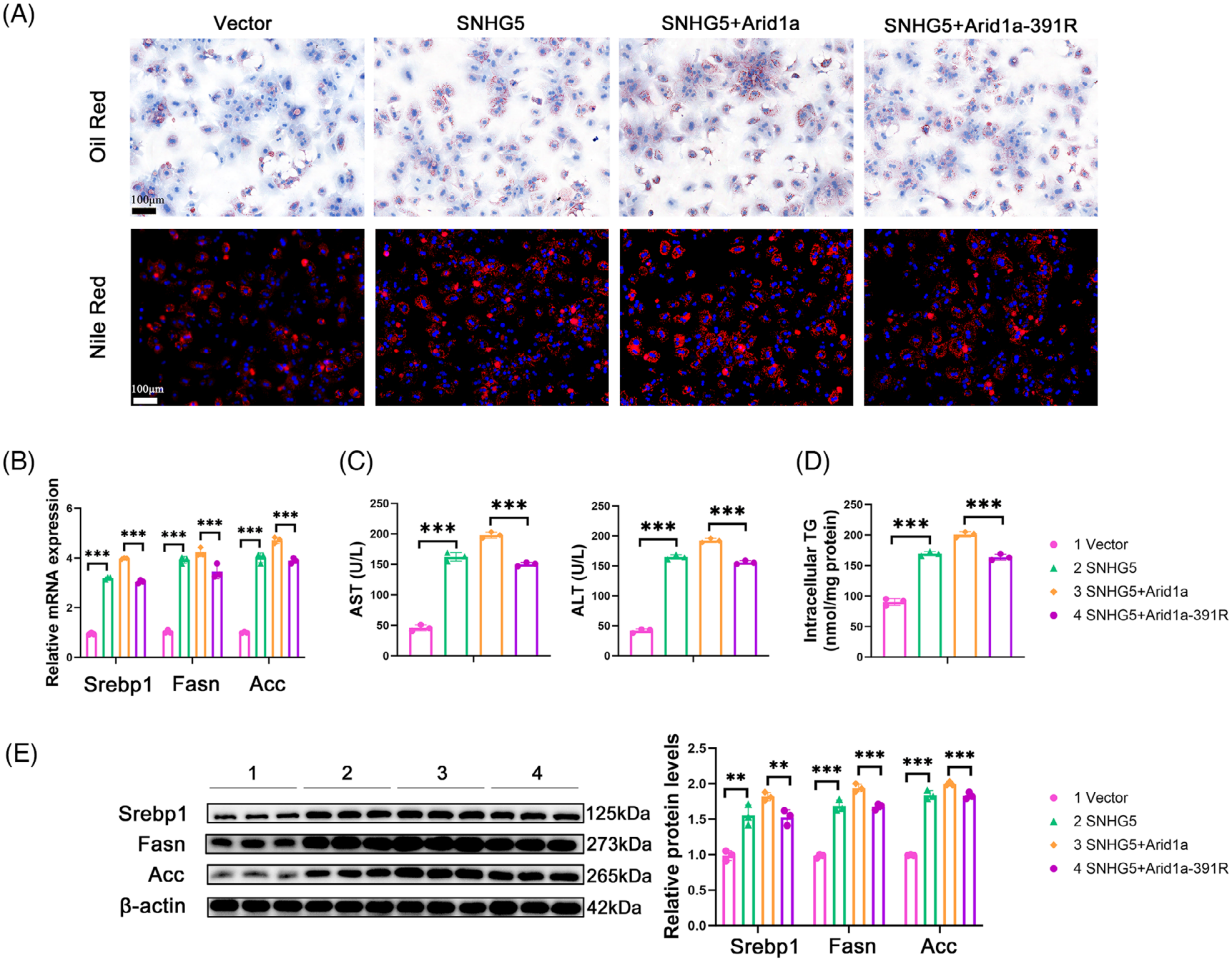
Small nucleolar RNA host gene 5 (SNHG5) promotes lipid synthesis by promoting Arid1a lactylation at K391. (A) Oil Red O and Nile Red staining in primary hepatocytes. (B and E) mRNA and protein expression levels of Srebp1, Fasn and Acc in primary hepatocytes. (C) Aspartate aminotransferase (AST) and alanine aminotransferase (ALT) levels in the culture supernatant of primary hepatocytes. (D) Intracellular triglyceride (TG) levels in primary hepatocytes. *n* = 3, ^**^ *p* < .01, ^***^ *p* < .001.

Given that lactylome analysis identified K391 as the primary lactylation site of Arid1a regulated by SNHG5, we next determined the functional role of this site in SNHG5‐mediated MAFLD progression. Phenotypically, Oil Red O and Nile Red staining demonstrated that the Arid1a‐K391R mutation markedly reversed SNHG5‐induced lipid droplet accumulation (Figure 6A). At the molecular and biochemical levels, Arid1a‐K391R significantly abrogated the SNHG5‐induced upregulation of fatty acid synthesis‐related genes at the mRNA (Figure 6B) and protein levels (Figure 6E). Correspondingly, the Arid1a‐K391R group exhibited substantial reductions in SNHG5‐mediated elevations of AST and ALT (Figure 6C) and intracellular TG content (Figure 6D) compared with the Arid1a‐WT group. In summary, these results demonstrate that lactylation of Arid1a at the K391 residue is an indispensable molecular event for SNHG5 to promote liver lipid synthesis and MAFLD progression in vitro.

### 3.7 SNHG5 promotes Arid1a lactylation through P300 and HBO1

**FIGURE 7:**
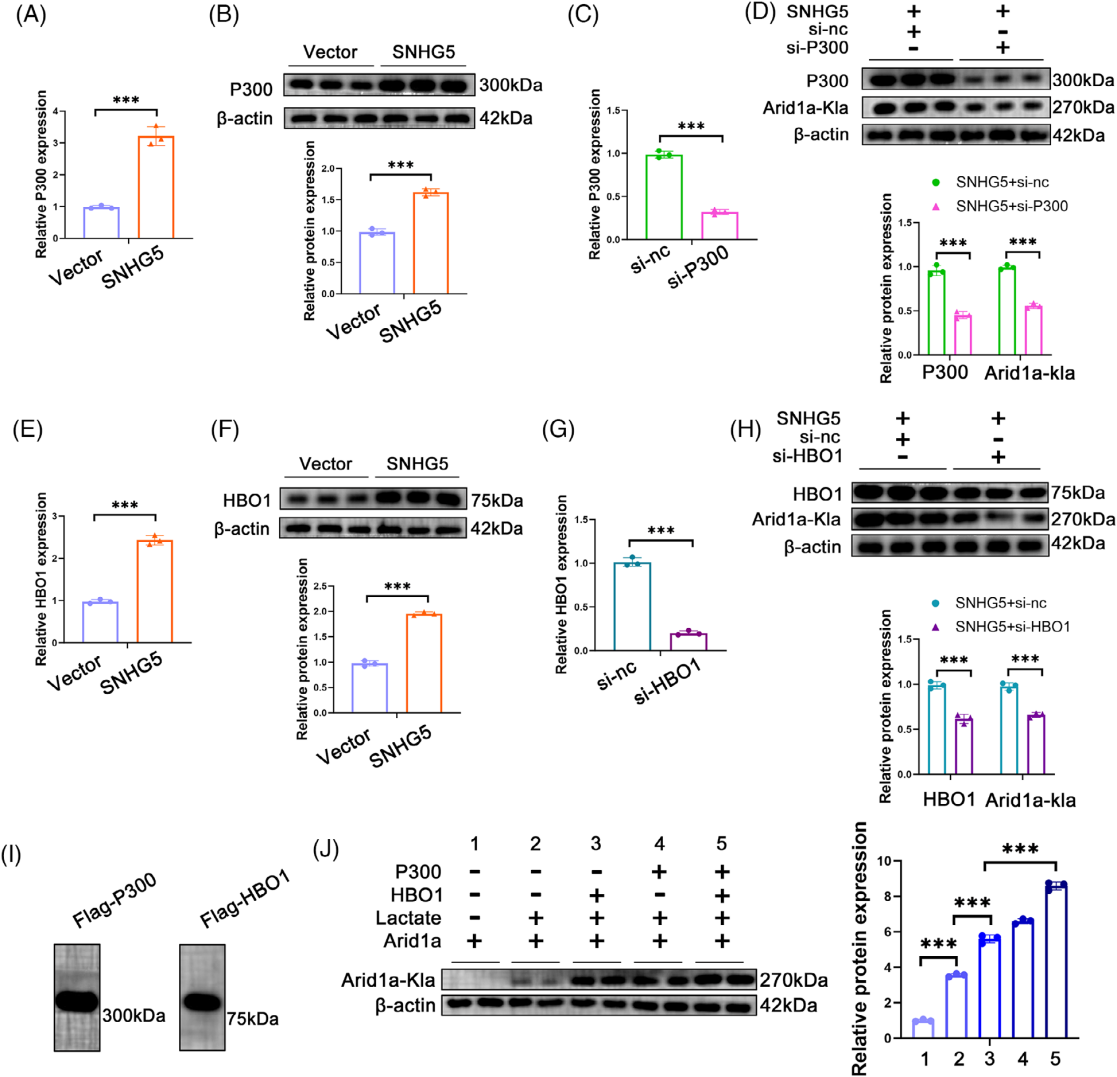
Small nucleolar RNA host gene 5 (SNHG5) promotes Arid1a lactylation through P300 and HBO1. (A–C) P300 mRNA and protein expression in primary hepatocytes. (D) P300 and Arid1a‐Kla protein expression in primary hepatocytes. (E–G) HBO1 mRNA and protein expression in primary hepatocytes. (H) HBO1 and Arid1a‐Kla protein expression in primary hepatocytes. (I) P300 and HBO1 protein expression. (J) Arid1a‐Kla protein expression in in vitro lactylation assays. *n* = 3, ^***^ *p* < .001.

Previous studies have reported that protein lactylation is catalysed by lysine acyltransferases, with P300, a member of the KAT family, being extensively characterised in this process.^29^ We therefore investigated the role of P300 in SNHG5‐mediated Arid1a lactylation. SNHG5 overexpression significantly upregulated P300 at the mRNA and protein levels (Figure 7A,B). To assess its functional contribution, loss‐of‐function experiments were performed by knocking down P300 in SNHG5‐overexpressing primary hepatocytes. Arid1a lactylation was partially reduced, but not abolished, by P300 knockdown (Figure 7C,D), suggesting that additional acyltransferases may contribute to Arid1a lactylation.

We next examined HBO1, a multifunctional lysine acyltransferase recently shown to catalyse histone lactylation.^30^ Similar to P300, SNHG5 overexpression significantly increased HBO1 expression at the mRNA (Figure 7E) and protein levels (Figure 7F). Importantly, knockdown of HBO1 in SNHG5‐overexpressing cells markedly inhibited Arid1a lactylation (Figure 7G,H), indicating a critical role for HBO1 in this process. To directly confirm that P300 and HBO1 catalyse Arid1a lactylation, recombinant P300 and HBO1 were expressed and purified (Figure 7I). In vitro lactylation assays using recombinant Arid1a as a substrate, with or without lactate supplementation, demonstrated that both P300 and HBO1 directly catalyse Arid1a lactylation and that this activity was further enhanced in the presence of lactate (Figure 7J). These results indicate that SNHG5 promotes Arid1a lactylation at least in part by upregulating the expression of P300 and HBO1.

### 3.8 SNHG5 promotes MAFLD progression via MCT1 in vivo

**FIGURE 8:**
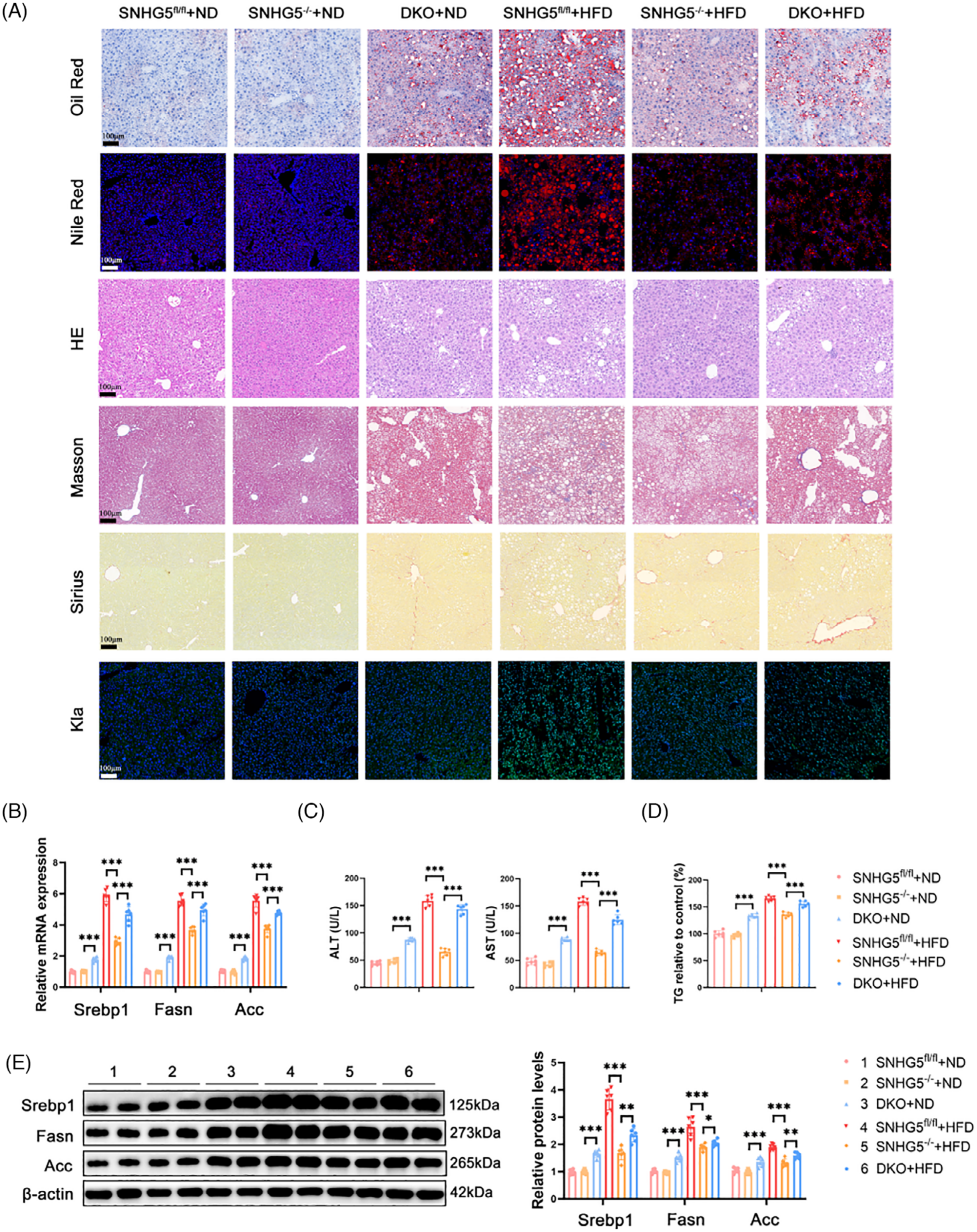
Small nucleolar RNA host gene 5 (SNHG5) promotes metabolic dysfunction‐associated fatty liver disease (MAFLD) progression via MCT1 in vivo. (A) Oil Red O, Nile Red, haematoxylin‒eosin (HE), Masson, Sirius Red staining and Kla levels in liver tissues. (B and E) mRNA and protein expression levels of Srebp1, Fasn and Acc in liver tissues. (C) Serum aspartate aminotransferase (AST) and alanine aminotransferase (ALT) levels. (D) Liver triglyceride (TG) content relative to control in liver tissues. *n* = 6, ^*^ *p* < .05, ^**^ *p* < .01, ^***^ *p* < .001.

To investigate whether SNHG5 influences MAFLD progression through MCT1 in vivo, hepatocyte‐specific SNHG5‐knockout (SNHG5^–/–^) mice and hepatocyte‐specific SNHG5 and MCT1 DKO mice were generated (Figure S6A) and subjected to ND or HFD. Histological analyses revealed that *SNHG5^–/–^* mice exhibited markedly reduced lipid droplet accumulation, as shown by Oil Red O and Nile Red staining, compared with SNHG5^fl/fl^ control mice under HFD conditions. This protective phenotype was largely reversed in DKO mice (Figure 8A). HE, Masson and Sirius Red staining further confirmed that SNHG5 deficiency significantly alleviated hepatic steatosis, inflammation and fibrosis in HFD‐fed mice, whereas these improvements were abrogated in DKO mice (Figure 8A). Immunofluorescence staining for pan‐lactylation (Kla) indicated that the reduced lactate levels observed in *SNHG5^–/–^* livers were restored in DKO mice (Figure 8A).

At the molecular level, downregulation of fatty acid synthesis‐related genes in *SNHG5^–/–^* livers was reversed in DKO mice under HFD (Figure 8B,E). Correspondingly, the elevations of liver injury markers (ALT and AST; Figure 8C) and liver TG content (Figure 8D) in HFD‐fed *SNHG5^fl/fl^* mice were significantly blunted in *SNHG5^–/–^* mice but restored in DKO mice. In summary, these in vivo results demonstrate that SNHG5 promotes MAFLD progression by regulating lactate metabolism through MCT1, and that loss of MCT1 reverses the protective effects of SNHG5 deficiency in the liver.

### 3.9 Validation of key findings in THLE‐2 human hepatocytes

To confirm the translational relevance of our findings in a human hepatocyte model, key in vitro experiments were performed in THLE‐2 cells, a well‐characterised human liver epithelial cell line. First, the role of SNHG5 in lipid metabolism was validated. Knockdown of SNHG5 using two independent siRNAs (si‐SNHG5‐1 and si‐SNHG5‐2) was confirmed by qRT‐PCR (Figure S7A). In PO‐treated THLE‐2 cells, SNHG5 knockdown significantly attenuated the increase in AST level (Figure S7B) and intracellular TG content (Figure S7C). Consistently, the PO‐induced upregulation of fatty acid synthesis‐related genes was markedly abrogated by SNHG5 knockdown (Figure S7D). Similarly, in THLE‐2 cells, RIP assays using the same anti‐MCT1 antibodies also demonstrated specific enrichment of SNHG5 (Figure S7E). Overexpression of SNHG5 in THLE‐2 cells increased AST levels (Figure S7F) and intracellular TG content (Figure S7G), both of which were reversed by co‐overexpression of MCT1. Importantly, SNHG5‐induced lactate accumulation was also blocked by MCT1 overexpression (Figure S7H), confirming that MCT1 is a critical mediator of SNHG5‐driven lactate accumulation. Consistent with the results in primary hepatocytes, the Arid1a‐K391R mutation significantly reduced Arid1a‐Kla levels in THLE‐2 cells overexpressing SNHG5 (Figure S7I). Finally, the role of acetyltransferases P300 and HBO1 in SNHG5‐mediated Arid1a lactylation was confirmed. SNHG5 overexpression significantly upregulated both P300 (Figure S7J) and HBO1 (Figure S7L) protein expression in THLE‐2 cells. Knockdown of either P300 (Figure S7K) or HBO1 (Figure S7M) in SNHG5‐overexpressing THLE‐2 cells markedly reduced Arid1a‐Kla levels, consistent with the observations in primary mouse hepatocytes. These results in human THLE‐2 cells confirm that the key regulatory mechanisms identified in mouse models—SNHG5 promoting lipid synthesis via MCT1‐dependent lactate accumulation and subsequent Arid1a lactylation mediated by P300 and HBO1—are conserved in human hepatocytes, supporting the translational potential of these findings.

## 4 DISCUSSION

Although MAFLD is prevalent, effective pharmacological treatments for this disease remain limited. A defining feature of MAFLD is hepatic steatosis, which is tightly regulated by lipogenic genes including Srebp‐1, Fasn and Acc.^31^, ^32^ Accordingly, strategies that suppress the expression of genes responsible for hepatic steatosis or lipid synthesis are critical for preventing both the initiation and progression of the disease. In this study, using hepatocyte‐specific SNHG5‐knockout and SNHG5/MCT1 DKO mouse models of MAFLD, we identified a previously unrecognised mechanism whereby SNHG5 promotes disease progression through regulation of Arid1a K391 lactylation via MCT1. This work identifies SNHG5 as a promising therapeutic candidate against MAFLD.

SNHG5, an lncRNA, has been implicated across multiple diseases. Previous studies have demonstrated its involvement in tumour proliferation, migration, and fibrosis through distinct molecular pathways. For example, Zhao et al. reported that the SNHG5/miR‐32 axis regulates gastric cancer progression by targeting KLF4,^33^ whereas Li et al. found that SNHG5 promotes HCC cell proliferation via UPF1 and the Wnt/β‐catenin signalling pathways.^34^ Consistent with these observations, our previous work showed that SNHG5 facilitates HSC activation and liver fibrosis through interaction with NF2.^11^ Building on these findings, the present study reveals, for the first time, a critical role of SNHG5 in metabolic liver disease, specifically in regulating lipid metabolism through lactylation modifications in MAFLD.

MCT1, encoded by the SLC16A1 gene, is a proton‐coupled MCT responsible for transmembrane lactate transport and is essential for maintaining lactate homeostasis in hepatocytes.^35^ Lactate functions not only as a metabolic substrate but also as a critical signalling molecule in hepatocytes, and its dynamic balance is indispensable for normal hepatic physiology.^36^ Reduced expression or impaired function of MCT1 directly blocks lactate efflux, leading to intracellular lactate accumulation.^37^ Our results demonstrated that upregulation of SNHG5 in hepatocytes markedly suppressed MCT1 expression and induced intracellular lactate accumulation. The attenuated steatotic difference observed between DKO and control mice under HFD, relative to ND, reflects the opposing effects of MCT1 loss and SNHG5 deletion, which are context‐dependent on basal versus upregulated SNHG5 expression under these dietary conditions. Notably, MCT1 exhibits distinct functions in hepatocytes and HSCs. In hepatocytes, MCT1 primarily mediates lactate export, whereas increased MCT1 expression in HSCs promotes intracellular lactate accumulation.^38^ This indicates that, during MAFLD progression, lactate produced by hepatocytes may be taken up by HSCs through intercellular communication, thereby influencing HSC activation and fibrosis. When MCT1‐mediated lactate efflux is impaired in hepatocytes, lactate accumulates intracellularly and may affect the metabolism and function of neighbouring HSCs via paracrine signalling. These findings indicate that MCT1 exerts complex metabolic regulatory functions between hepatic parenchymal and non‐parenchymal cells, and that its cell‐specific mechanisms in MAFLD warrant further investigation. Furthermore the divergent metabolic context and intervention duration explain the inconsistent phenotypes between our work and prior research.^39^ That study applied transient siRNA‐mediated MCT1 silencing in mice fed standard chow, where acute AMPK activation inhibited lipogenesis and mitigated lipid deposition. By contrast, our HFD‐induced MAFLD model involves stable, long‐term modulation of SNHG5. Pathologically upregulated SNHG5 persistently suppresses MCT1, causing sustained intracellular lactate accumulation thereby aggravating hepatic steatosis.

Lactylation is a recently identified PTM implicated in tumourigenesis, metabolism and immune responses.^36^ Previous studies have shown that lactylation contributes to MAFLD by modulating the activity of metabolic enzymes, thereby influencing both energy and lipid metabolism. A key novelty of the present study is the demonstration that SNHG5 promotes Arid1a lactylation at K391 by increasing intracellular lactate levels, which in turn exacerbates lipid deposition, liver injury and MAFLD progression. These results establish a direct connection between lactate metabolism and chromatin regulation, providing new insights into the molecular mechanisms underlying MAFLD.

Arid1a, a core component of the chromatin remodelling complex, is involved in cell proliferation, differentiation and regulation of metabolic gene expression. Recent studies have reported that Arid1a deficiency leads to insulin resistance, steatohepatitis and dysregulated lipid metabolism.^40^, ^41^ However, lactylation of Arid1a in the context of MAFLD has not been previously reported. The present study provides the first evidence that SNHG5 regulates Arid1a K391 lactylation via P300 and HBO1, representing a critical molecular event driving MAFLD progression. These findings expand the known regulatory functions of Arid1a in metabolic liver disease and identify a potential new target for therapeutic intervention in MAFLD.

This study has several limitations. First, the precise binding domain and the detailed molecular mechanism underlying the interaction between SNHG5 and MCT1 require further validation. Second, additional proteins or signalling pathways may also contribute to the regulation of the SNHG5‒Arid1a lactylation axis. Moreover, the crosstalk among SNHG5, hepatic lactate metabolism, and the gut microbiota remains to be explored. Notably, gut microbiota produce abundant lactate, which may act synergistically with host lactate metabolism to promote MAFLD progression.^42^

In conclusion, we demonstrate that SNHG5 induces MCT1‐mediated Arid1a K391 lactylation via P300 and HBO1, thereby promoting MAFLD. Although hepatocyte‐specific SNHG5 knockout markedly alleviates hepatic steatosis and metabolic disorders in mice, the translational application of SNHG5 as a therapeutic target still faces challenges, including delivery efficiency, safety, stability and off‐target effects. Future studies should focus on optimising liver‐targeted delivery systems and validating both the therapeutic efficacy and safety of SNHG5 inhibition, which may provide novel strategies for clinical intervention in MAFLD.

## AUTHOR CONTRIBUTIONS

*Writing—original draft, project administration and writing*: Xinmiao Li. *Methodology, investigation, formal analysis and data curation*: Feng Jiang and Binbo Fang. *Formal analysis and data curation*: Lifan Lin. *Conceptualisation and funding acquisition*: Tanzhou Chen and Jianjian Zheng.

## CONFLICT OF INTEREST STATEMENT

The authors declare no financial conflicts of interest or personal relationships that could have influenced the findings presented in this paper.

## ETHICS STATEMENT

All procedures involving human samples followed the principles of the Declaration of Helsinki and were approved by the Ethics Committee of the First Affiliated Hospital of Wenzhou Medical University (KY2022‐139) on 18 November 2021. The Laboratory Animal Ethics Committee of Wenzhou Medical University approved all animal experiments conducted in this study (no. wydw2024‐0443).

## Supporting information



FIGURE S1 Related to Figure 1. Characterisation of high‐fat diet (HFD)‐ and high‐fat, high‐cholesterol diet (HFHC)‐induced metabolic dysfunction‐associated fatty liver disease (MAFLD) mouse models. (A) Representative gross morphology of livers. (B) Liver weight. (C) Liver triglyceride (TG) content in liver tissues. (D) Haematoxylin‒eosin (HE) staining of liver tissues. (E) Serum alanine aminotransferase (ALT) and aspartate aminotransferase (AST) levels. *n* = 6, ^***^ *p *< .001.

TABLE S1. Primers used for quantitative real‐time PCR (qRT‐PCR) (mouse).

## Data Availability

The data utilised and/or analysed during this study are available from the corresponding author upon reasonable request.
