# Supplementary material for: Role of LncSNHG5 in MAFLD: Mechanisms of Arid1a K391 lactylation and lipid accumulation
Source: Clin Transl Med. 2026 Jul 24;16(7):e70740. doi: 10.1002/ctm2.70740 (PMC13400984; doi:10.1002/ctm2.70740)
Supplement: Supplementary file 1 — FIGURE S1 Related to Figure 1. Characterisation of high‐fat diet (HFD)‐ and high‐fat, high‐cholesterol diet (HFHC)‐induced metabolic dysfunction‐associated fatty liver disease (MAFLD) mouse models. (A) Representative gross morphology of livers. (B) Liver weight. (C) Liver triglyceride (TG) content in liver tissues. (D) Haematoxylin‒eosin (HE) staining of liver tissues. (E) Serum alanine aminotransferase (ALT) and aspartate aminotransferase (AST) levels. n = 6, *** p < .001. [file CTM2-16-e70740-s002.docx]

**The Role of LncSNHG5 in MAFLD: Mechanisms of Arid1a K391 Lactylation and Lipid Accumulation**

Xinmiao Li^1#^, Feng Jiang^2#^, Binbo Fang^2#^, Lifan Lin^3^, Jianjian Zheng^2,*^, Tanzhou Chen^4,*^

^1^ Department of Clinical Laboratory, Key Laboratory of Clinical Laboratory Diagnosis and Translational Research of Zhejiang Province, the First Affiliated Hospital of Wenzhou Medical University, Wenzhou, Zhejiang, China

^2^ Zhejiang Key Laboratory of Intelligent Cancer Biomarker Discovery and Translation, The First Affiliated Hospital of Wenzhou Medical University, Wenzhou, China

^3^ Department of Clinical Laboratory, The Third Affiliated Hospital of Wenzhou Medical University, Ruian, Zhejiang, China

^4^ Department of Gastroenterology and Hepatology, The First Affiliated Hospital of Wenzhou Medical University, Wenzhou, China.

^#^ These authors have contributed equally to this work.

***Correspondence:**

Tanzhou Chen, Department of Gastroenterology and Hepatology, The First Affiliated Hospital of Wenzhou Medical University, No.2 fuxue lane, Wenzhou, Zhejiang, PR China. E-mail: [cctzz@wmu.edu.cn](mailto:cctzz@wmu.edu.cn)

Jianjian Zheng, Zhejiang Key Laboratory of Intelligent Cancer Biomarker Discovery and Translation, The First Affiliated Hospital of Wenzhou Medical University, No.2 fuxue lane, Wenzhou, Zhejiang, P.R. China; E-mail: [zjj@wmu.edu.cn](mailto:zjj@wmu.edu.cn)

**Supporting Information**

**
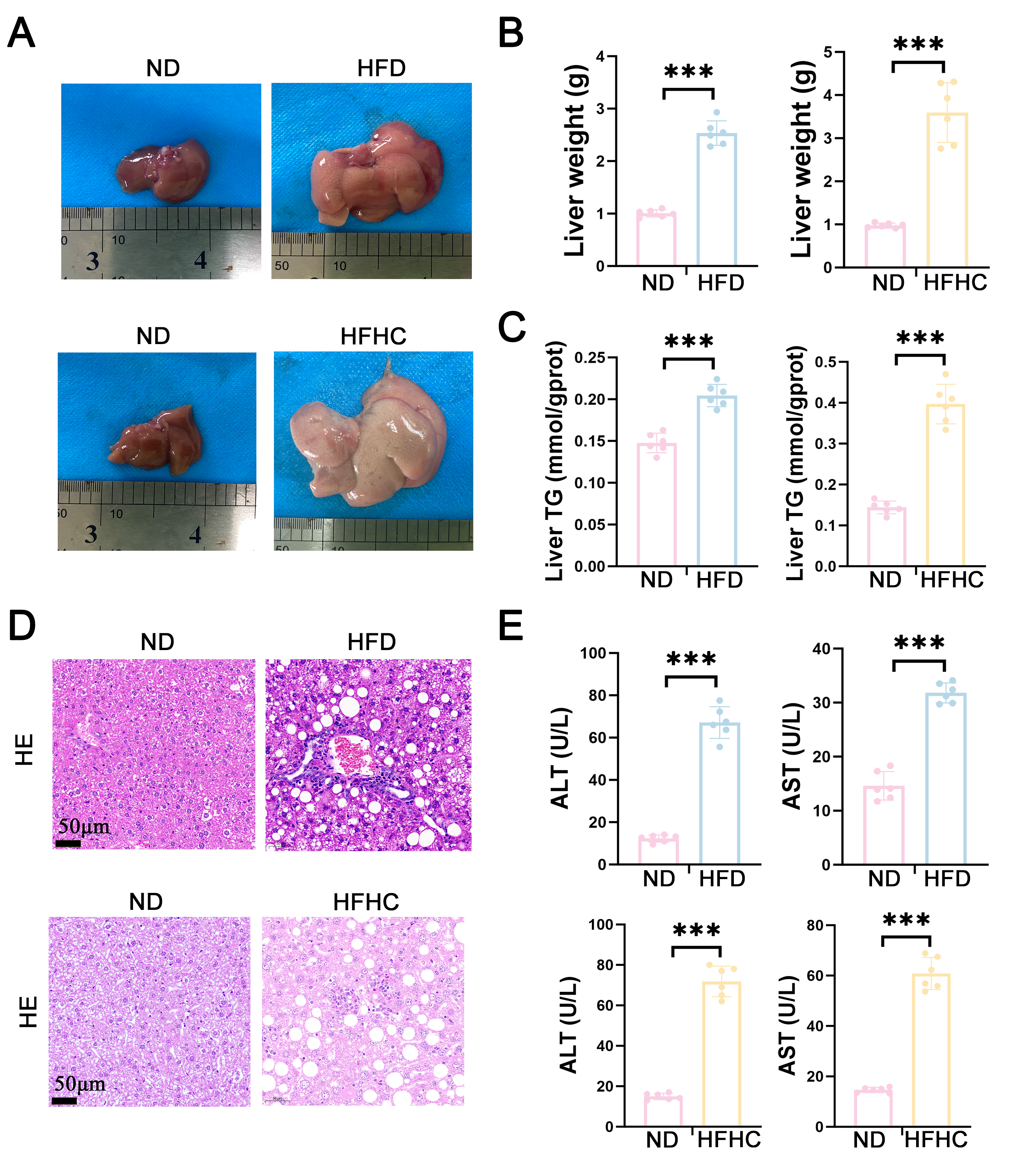
**

Figure S1 Related to Figure 1. Characterization of HFD and HFHC-induced MAFLD mouse models

(A) Representative gross morphology of livers. (B) Liver weight. (C) Liver TG content in liver tissues. (D) HE staining of liver tissues. (E) Serum ALT and AST levels. n=6, ****P*< 0.001.

**
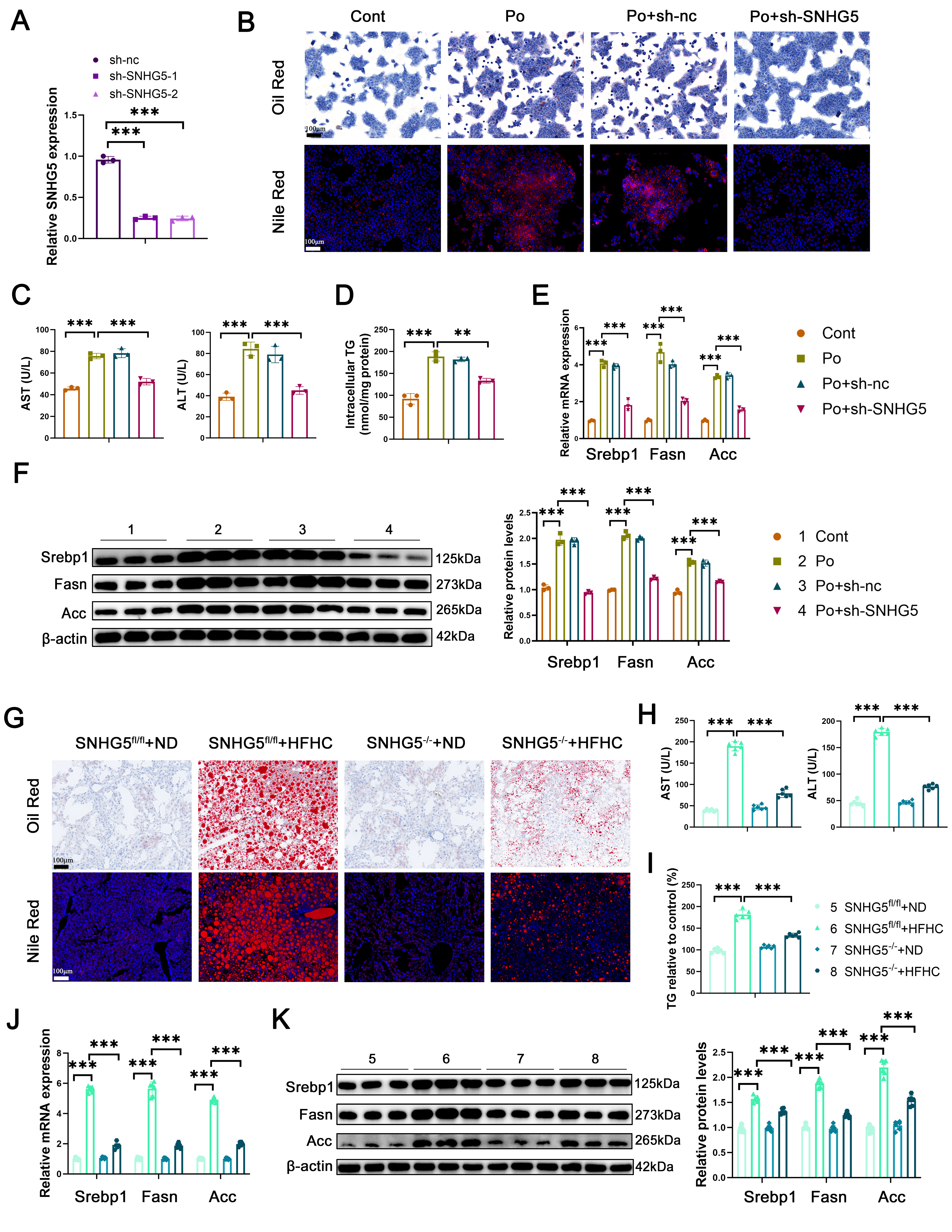
**

Figure S2 Related to Figure 2. SNHG5 deficiency inhibits lipid deposition in AML12 cells and HFHC-induced MAFLD mouse models

(A) qRT-PCR analysis of SNHG5 expression in AML12 cells (n=3). (B) Oil Red O and Nile Red staining in AML12 cells (n=3). (C) AST and ALT levels in the culture supernatant of AML12 cells (n=3). (D) TG levels in AML12 cells (n=3). (E and F) Srebp1, Fasn, and Acc mRNA and protein expression in AML12 cells (n=3). (G) Oil Red O and Nile Red staining in liver tissues (n=6). (H) Serum AST and ALT levels (n=6). (I) Liver TG content relative to control in liver tissues(n=6). (J and K) Srebp1, Fasn, and Acc mRNA and protein expression in liver tissues (n=6). ***P*< 0.01, ****P*< 0.001.


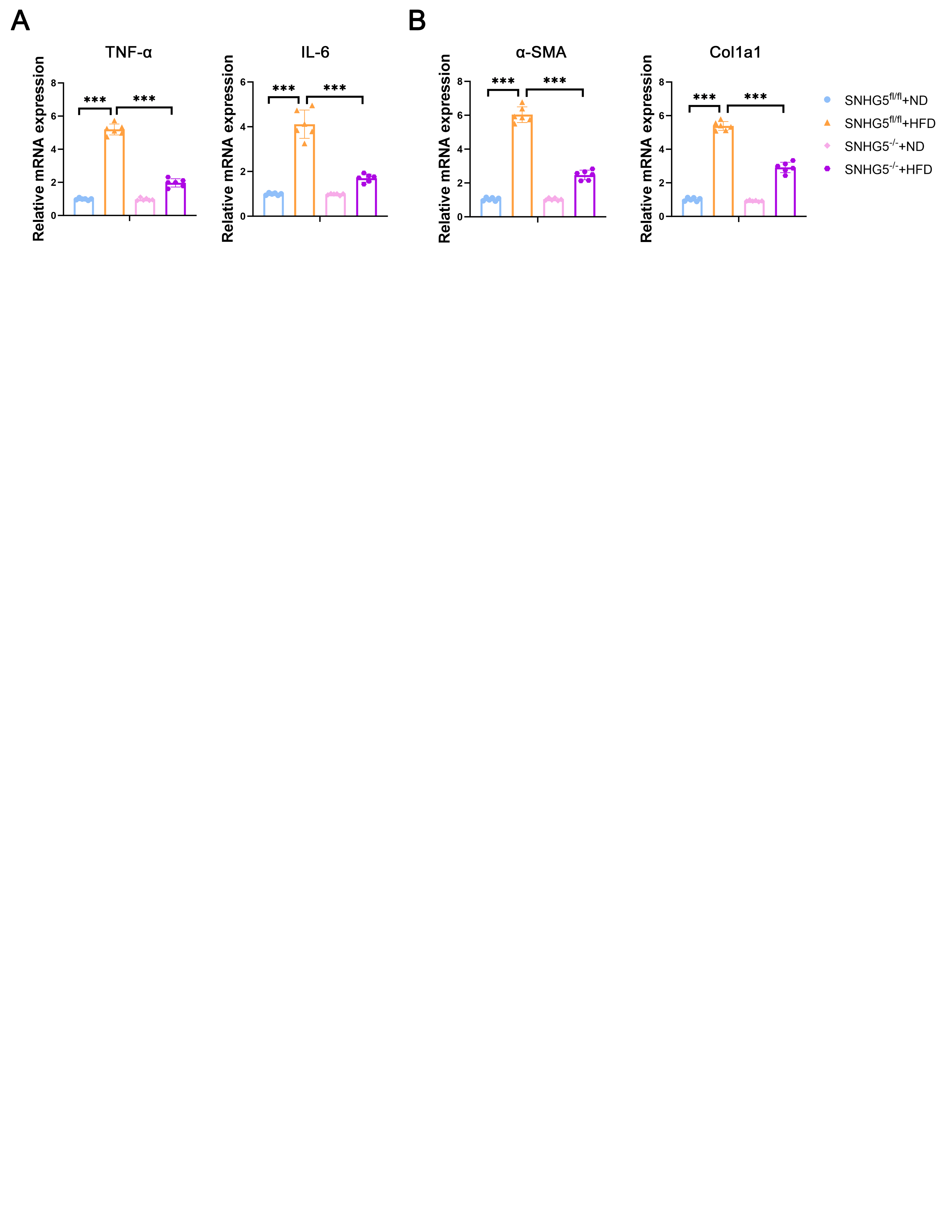


Figure S3 Related to Figure 2. SNHG5 deficiency reduces liver inflammatory and fibrotic responses in HFD-induced MAFLD mice

(A) qRT-PCR analysis of TNF-α and IL-6 mRNA expression in liver tissues. (B) qRT-PCR analysis of fibrotic marker (α-SMA, Col1a1) mRNA expression in liver tissues. n=6, ****P*< 0.001.


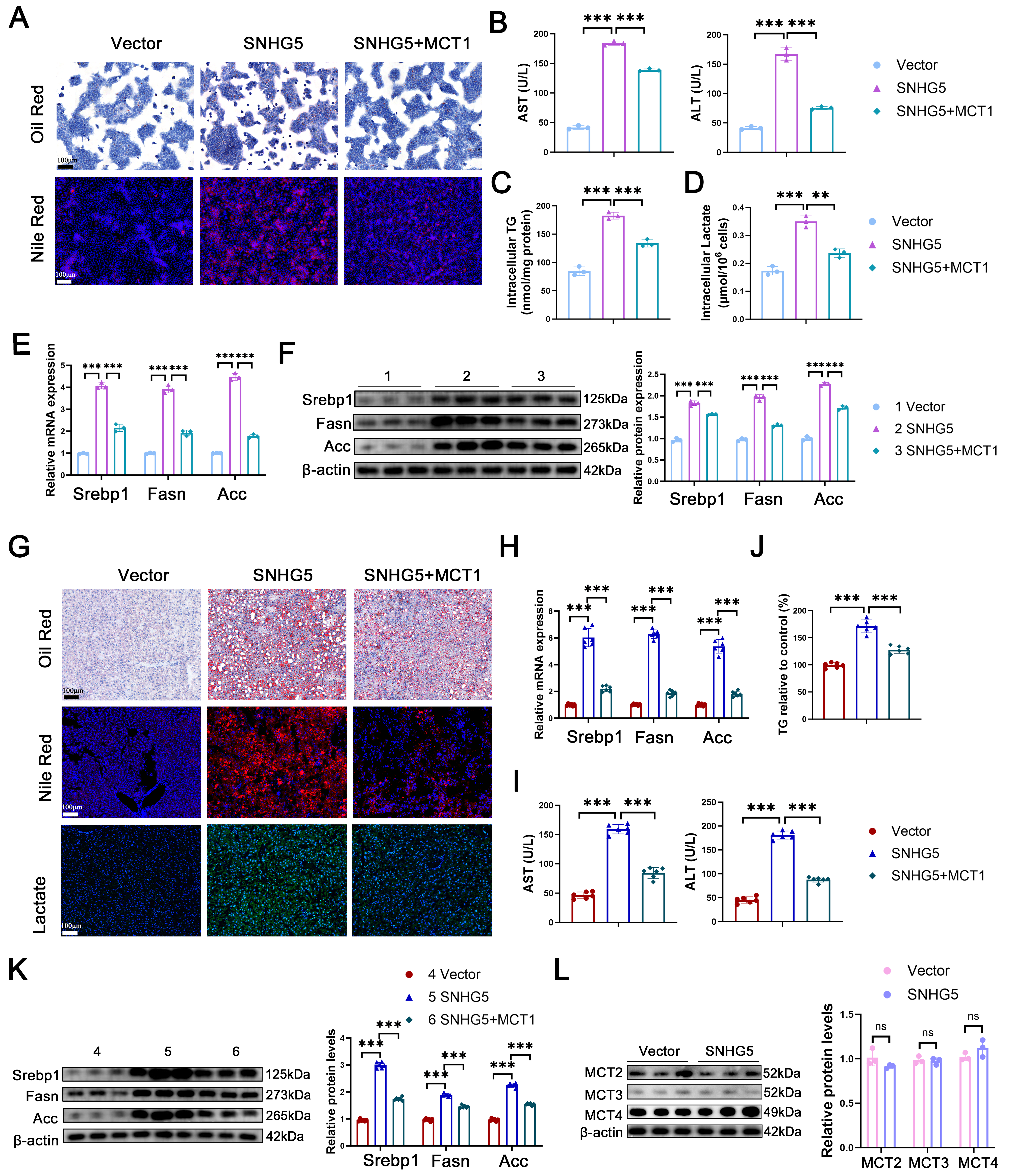


Figure S4 Related to Figure 3. SNHG5 promotes lipid accumulation by regulating lactate via MCT1 in AML12 cells and HFD-induced MAFLD mice, and shows specificity for MCT1

(A) Oil Red O and Nile Red staining in AML12 cells (n=3). (B) AST and ALT levels in the culture supernatant of AML12 cells (n=3). (C) Intracellular TG content in AML12 cells (n=3). (D) Intracellular lactate levels in AML12 cells (n=3). (E and F) qRT-PCR and Western blot analysis of Srebp1, Fasn, and Acc mRNA and protein expression in AML12 cells (n=3). (G) Oil Red O, Nile Red, and lactate staining in liver tissues from HFD-induced MAFLD mice (n=6). (H and K) qRT-PCR and Western blot analysis of Srebp1, Fasn, and Acc mRNA and protein expression in liver tissues from HFD-induced MAFLD mice (n=6). (I) Serum AST and ALT levels in HFD-induced MAFLD mice (n=6). (J) Liver TG content relative to control in liver tissues from HFD-induced MAFLD mice (n=6). (L) Western blot analysis of MCT2, MCT3, and MCT4 protein expression in AML12 cells (n=3). ***P*< 0.01, ****P*< 0.001, ns=no significance.


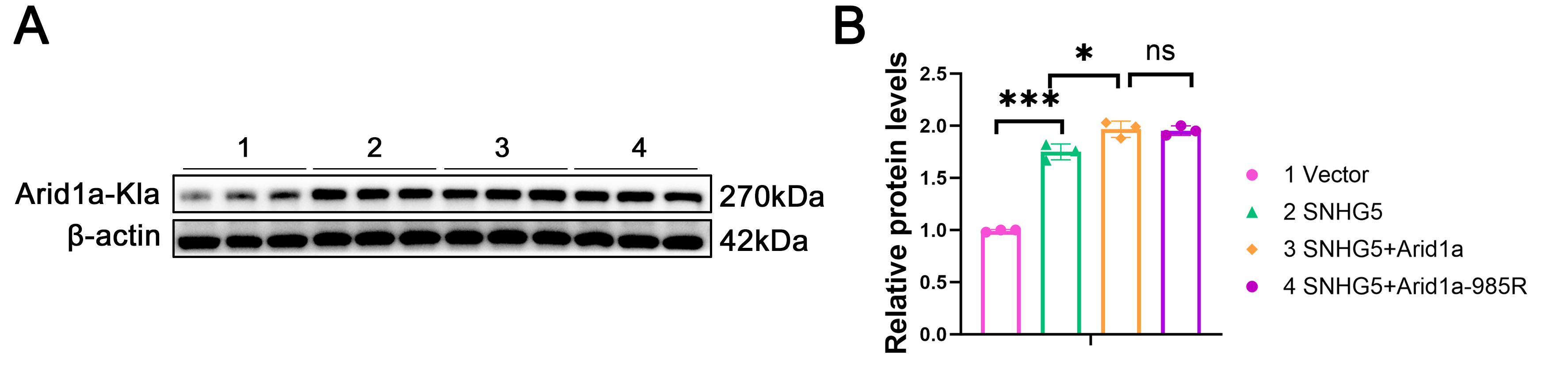


Figure S5 Related to Figure 5. Arid1a K985 mutation does not affect SNHG5-mediated Arid1a lactylation in primary hepatocytes

(A and B) Western blot analysis of Arid1a-Kla protein levels in primary hepatocytes. n=3, **P*< 0.05, ****P*< 0.001, ns=no significance.


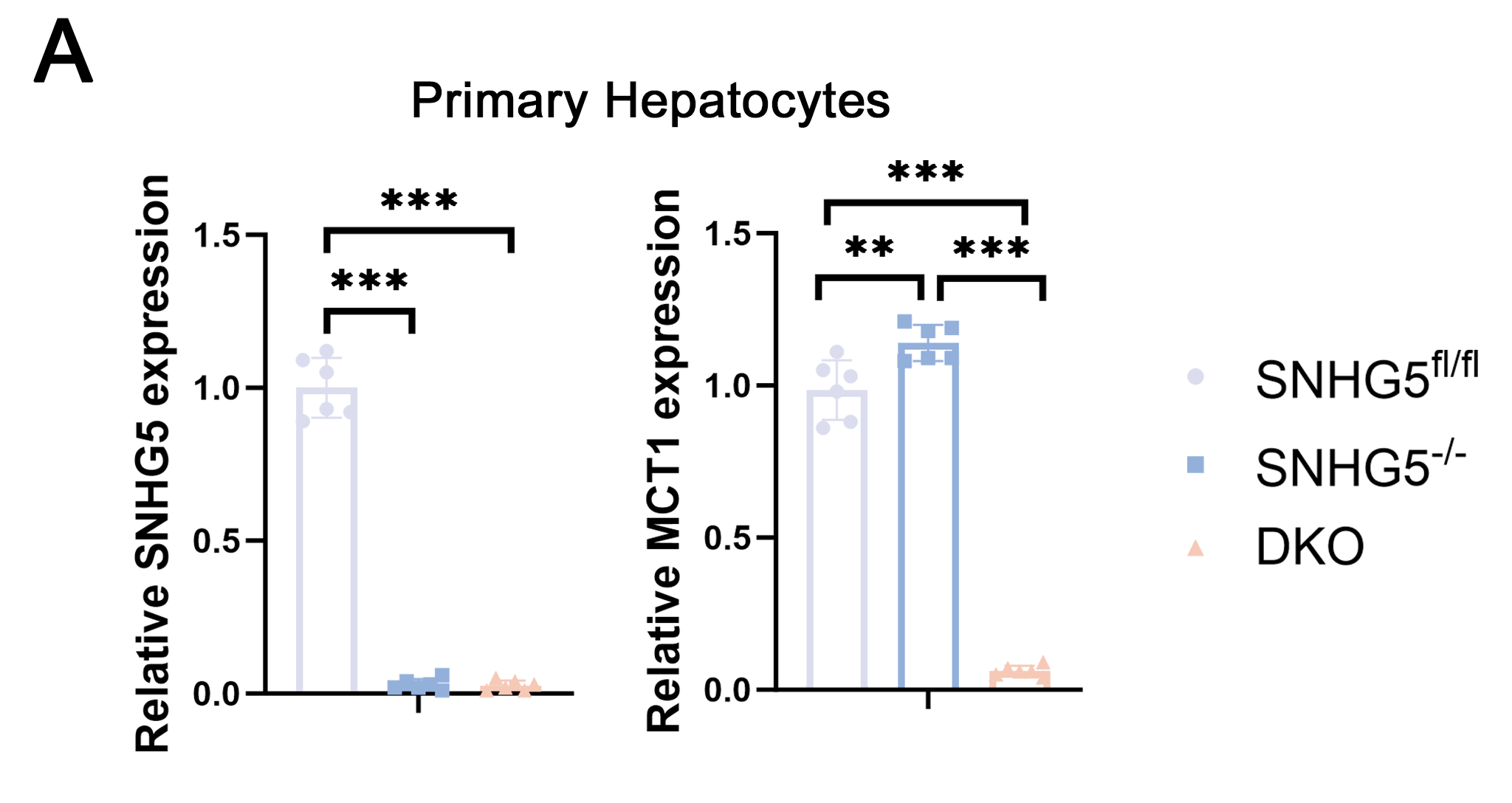


Figure S6 Related to Figure 8. Knockout efficiency in primary hepatocytes

(A) qRT-PCR analysis of SNHG5 and MCT1 expression in primary hepatocytes isolated from *SNHG5^fl/fl^*, *SNHG5^-/-^* and DKO mice. n=6, ***P*< 0.01, ****P*< 0.001.


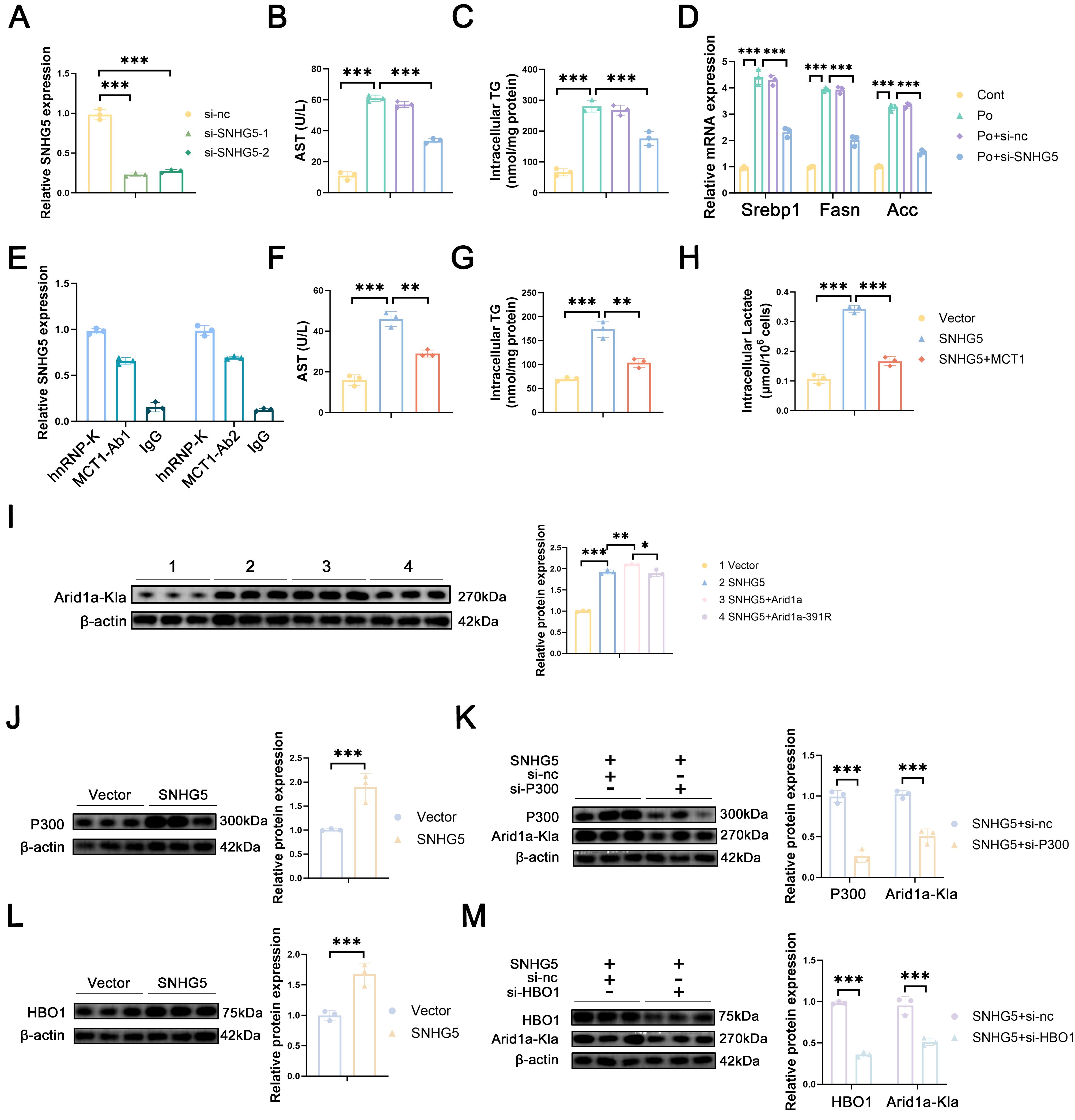


Figure S7. SNHG5-MCT1-lactate-P300/HBO1-Arid1a lactylation axis in THLE-2 human liver epithelial cells

(A) qRT-PCR analysis of SNHG5 expression in THLE-2 cells. (B) AST and ALT levels in the culture supernatant of THLE-2 cells. (C) AST and TG levels in THLE-2 cells. (D) qRT-PCR analysis of Srebp1, Fasn, and Acc mRNA expression in THLE-2 cells. (E) RIP experiments in THLE-2 cells using MCT1 antibody, with hnRNP-K antibody and IgG serving as positive and negative controls, respectively. (F and G) AST and TG levels in THLE-2 cells. (H) Intracellular lactate levels in THLE-2 cells. (I) Western blot analysis of Arid1a-Kla levels in THLE-2 cells. (J) Western blot analysis of P300 levels in THLE-2 cells. (K) Western blot analysis of P300 and Arid1a-Kla protein levels in THLE-2 cells. (L) Western blot analysis of HBO1 protein levels in THLE-2 cells. (M) Western blot analysis of HBO1 and Arid1a-Kla protein levels in THLE-2 cells. n=3, **P*< 0.05, ***P*< 0.01, ****P*< 0.001.

**Funding Statement**

This project was supported by the National Natural Science Foundation of China (No. 81873576), the Key Laboratory of Clinical Laboratory Diagnosis and Translational Research of Zhejiang Province (2022E10022), the Zhejiang Provincial Medical and Health Planning Project (No.2025KY102), the Major Projects of Wenzhou Science and Technology Bureau (ZY2024005), and the Joint Project of the Zhejiang Provincial Natural Science Foundation (LBY24H200005)
