# Supplementary material for: Role of LncSNHG5 in MAFLD: Mechanisms of Arid1a K391 lactylation and lipid accumulation
Source: Clin Transl Med. 2026 Jul 24;16(7):e70740. doi: 10.1002/ctm2.70740 (PMC13400984; doi:10.1002/ctm2.70740)
Supplement: Supplementary file 2 — TABLE S1. Primers used for quantitative real‐time PCR (qRT‐PCR) (mouse). [file CTM2-16-e70740-s001.docx]

| Table S1. The primers used for qRT-PCR (Mouse) | | |
| --- | --- | --- |
| **Primer** | **Sense** | **Antisense** |
| SNHG5 | CTTTTACGTCGGCCTTCGCGAGCGTCTGGG | TTAGTGGATTTTCCATTTAATGCTCCCCAT |
| Srebp1 | CCAGGTGACCCGGCTATTC | CGGCACCCGCTGCTTTA |
| Fasn | AACCGGCTCTCCTTCTTCTT | TTGGGCTTCAGCAGGACATT |
| Acc | ACAGTGGAGCTAGAATTGGAC | ACTTCCCGACCAAGGACTTTG |
| Pfkm | ATGACCCATGAAGAGCACCA | GCACCGGTGAAGATACCAAC |
| Gpi | TCGCCCAACTCTATTG | GATGCCCTGAAGAT |
| Hif1a | TTCCCGACTAGGCCCATTC | CAGGTATTCAAGGTCCCATTTCA |
| Gck1 | GAGATGGATGTGGTGGCAAT | ACCAGCTCCACATTCTGCAT |
| Pkm | CCACTTGCAATTATTTGAGGAA  5′-GGTGACTCCGTCGCAATTAT-3′ | GTGAGCAGACCTGCCAGACT  5′-CGTCGAACAAAGCACAGAAA-3′ |
| Pgm2 | TGAAGACATTGTCCGCCAGCAC | CTGCAGTTGCGTCTACGTTCTC |
| Pfkl | CTACGAGGGCTATGAGGGC | GATGACGCACAGGTTGGTGA |
| Slc16a1 | TGTAGGTGCAGCAGCCAAG | TTGAAAGCAAGCCCAAGACC |
| Pck2 | GGGTGCTAG ACTGGATCTGC | CTG GTT GAC CTG CTC TGTCA |
| Hk2 | GAGCCACCACTCACCCTACT | CCAGGCATTCGGCAATGTG |
| P300 | GCCAAGTATGCCAACCCTAA | TGTTCATTTGCTGAGCTTGG |
| HBO1 | AAAAGTTGGCTCCCCAGAAC | GCCGTCTCCTGACTGATTTC |
| β-actin | GGCTGTATTCCCCTCCATCG | CCAGTTGGTAACAATGCCATGT |

| TableS2. Antibody for Western Blot. | | |
| --- | --- | --- |
| **Antibodies** | **Source** | **Identifier** |
| Mct1 | Thermo Fisher | PA5-72957 |
| Pck2 | Abcam | Ab187145 |
| Hk2 | Abcam | Ab209847 |
| Pkm | Abcam | Ab150377 |
| Pgm2 | Abcam | Ab151746 |
| Pfkl | Abcam | Ab97443 |
| Pfkm | Abcam | Ab232495 |
| Gpi | Proteintech | 15171-1-AP |
| Hif1a | Abcam | Ab308433 |
| Gck1 | Abcam | Ab88056 |
| Arid1a | Abcam | Ab182560 |
| P300 | Thermo Fisher | 33-7600 |
| Hbo1 | Abcam | Ab190908 |
| Mct2 | Affinity | DF9633 |
| Mct3 | Affinity | DF13644 |
| Mct4 | Affinity | AF5253 |
| β-actin | Abcam | Ab6276 |

**TableS3. Arid1a Protein Lactylation site**

| **No.** | **Protein Name** | **Lactylation site** | **SNHG5/Cont** | ***P* value** |
| --- | --- | --- | --- | --- |
| 1 | Arid1a | (K391) | 6.689251881 | 0.005254392 |
| 2 | Arid1a | (K985) | 2.126210503 | 0.027807015 |
| 3 | Arid1a | (K981,M982) | 1.076037223 | 0.21090809 |
| 4 | Arid1a | (K981) | 1.460272929 | 0.162016061 |
| 5 | Arid1a | (M1614,K1616) | 0.94276767 | 0.38808285 |
| 6 | Arid1a | (M1614,K1616,M1635) | 0.891781881 | 0.584467941 |
| 7 | Arid1a | (K1616) | 1.099829244 | 0.467516764 |
| 8 | Arid1a | (K1202) | 0.892716802 | 0.709347163 |
| 9 | Arid1a | (M982,K985) | 0.696048209 | 0.112987418 |
| 10 | Arid1a | (K1809) | 1.234149889 | 0.191001568 |
| 11 | Arid1a | (C1825,K1828) | 0.751499929 | 0.399659298 |
| 12 | Arid1a | (M1225,K1231) | 0.890313304 | 0.628551995 |
| 13 | Arid1a | (K1926) | 0.954066727 | 0.853312919 |
